# Solubilised bright blue-emitting iridium complexes for solution processed OLEDs†
†Electronic supplementary information (ESI) available: Synthesis CIF of the crystal structures. CCDC 1440897–1440899. For ESI and crystallographic data in CIF or other electronic format see DOI: 10.1039/c6tc00151c



**DOI:** 10.1039/c6tc00151c

**Published:** 2016-02-11

**Authors:** Adam F. Henwood, Ashu K. Bansal, David B. Cordes, Alexandra M. Z. Slawin, Ifor D. W. Samuel, Eli Zysman-Colman

**Affiliations:** a Organic Semiconductor Centre , EaStCHEM School of Chemistry , University of St Andrews , St Andrews , Fife KY16 9ST , UK . Email: eli.zysman-colman@st-andrews.ac.uk ; http://www.zysman-colman.com ; Fax: +44 (0)1334 463808 ; Tel: +44 (0)1334 463826; b Organic Semiconductor Centre , SUPA , School of Physics and Astronomy , University of St Andrews , Fife , KY16 9SS , UK

## Abstract

Combining a sterically bulky, electron-deficient 2-(2,4-difluorophenyl)-4-(2,4,6-trimethylphenyl)pyridine (dFMesppy) cyclometalating C^∧^N ligand with an electron rich, highly rigidified 1,1′-(α,α′-*o*-xylylene)-2,2′-biimidazole (*o*-xylbiim) N^∧^N ligand gives an iridium complex, [Ir(dFMesppy)_2_(*o*-xylbiim)](PF_6_), that achieves extraordinarily bright blue emission (*Φ*_PL_ = 90%; *λ*_max_ = 459 nm in MeCN) for a cationic iridium complex. This complex is compared with two reference complexes bearing 4,4′-di-*tert*-butyl-2,2′-bipyridine, and solution-processed organic light emitting diodes (OLEDs) have been fabricated from these materials.

## Abstract 2

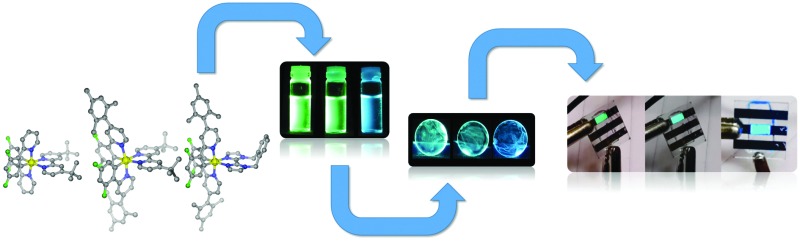
Combining a sterically bulky, electron-deficient cyclometalating C^∧^N ligands with an electron rich, highly rigidified N^∧^N ligand gives an iridium complex, that achieves extraordinarily bright blue emission (*Φ*_PL_ = 90%; *λ*_max_ = 459 nm in MeCN) for a cationic iridium complex.

## Introduction

The world's ever increasing demand for energy necessitates an overhaul in virtually all areas of technology. One of the most pressing areas of concern in this problem is that of artificial lighting, which constitutes approximately 19% of total global electricity consumption.^1^ This tremendous electricity consumption and its problematic societal and environmental consequences stem from the poor efficiencies of commonly used lighting technologies: incandescent light bulbs are typically only 5% efficient, and even compact fluorescent lighting enforced by the EU is only about 20% efficient.^1^ There is thus an acute need for new lighting technologies that can couple high lighting efficiencies with low fabrication cost.

Organic light emitting devices (OLEDs) have been studied for some time as one of the leading technologies for meeting these challenges. When iridium-based complexes are used as the emissive materials, very high device efficiencies are possible, since these complexes can harvest both singlet and triplet excitons generated from electron–hole recombination by their strong spin–orbit coupling effects.^2^ Normally these devices are comprised of a number of individual layers, which serve to carry out a single function, such as charge transport or emission. They are frequently fabricated by vacuum sublimation of sequentially deposited individual layers at sufficiently high temperatures and low pressures.^3^ Although processing in this fashion has been shown to give devices with impressive performance metrics, drawbacks such as the considerable production costs and poor scalability of this fabrication method has impeded it from being adopted on an industrial scale for lighting.^4^ Furthermore, the high temperatures associated with this technique mean only some materials are suitable, and there are reports of thermal isomerisation of these iridium complexes during device fabrication that can impact the overall device performance.^5^ Solution processing addresses these issues; plastic electronics can be printed onto a flexible substrate in a roll-to-roll fashion.^6^ However, typically the efficiencies of devices processed from solution are lower than their counterparts fabricated by vacuum methods.^4a,7^

With respect to the performance of iridium-based emitters in OLEDs, a similar problem exists. Although exceptional performance red^2*b*^ and green^8^ OLEDs based on iridium have been reported, a corresponding deep blue emitter remains elusive.^2*a*^ This is due in no small part to the host–guest configuration of the emissive layers in OLEDs – the requirement thus being that the high triplet energies required for blue emitters (>2.8 eV) necessitate even higher triplet energies for the host materials. At these energy regimes, realising triplet host energies that are compatible with the emitter and suitable functional device materials (charge transport layers, electrodes) has become increasingly difficult to achieve. Thus, even devices that show deep blue emission using iridium complexes show poorer efficiencies compared to their red- and green-emitting counterparts (∼30% EQE); a recent review on blue emitters in OLEDs identified a champion true blue device based on iridium as having CIE coordinates of (0.14, 0.10), with an EQE of 7.6%,^2*a*^ while a recent report^9^ outlined the use of a tris-cyclometalated NHC iridium complex that achieved CIE coordinates of (0.16, 0.09) at an EQE of 10.1% – a different story from the higher efficiencies (>20%) reported for sky-blue emitters.^2a,10^

The problem of attaining bright blue emission for any iridium complex stems from the increasing likelihood of thermal population of non-emissive metal-centred (MC) states as the optical gap increases. This problem is particularly pertinent for cationic emitters, since their rational design prohibits employing a third anionic strong field cyclometalating ligand to efficiently destabilise the MC states. Instead, a combination of highly electron deficient anionic C^∧^N ligands, and electron rich neutral N^∧^N ligands are required.

**Fig. 1:**
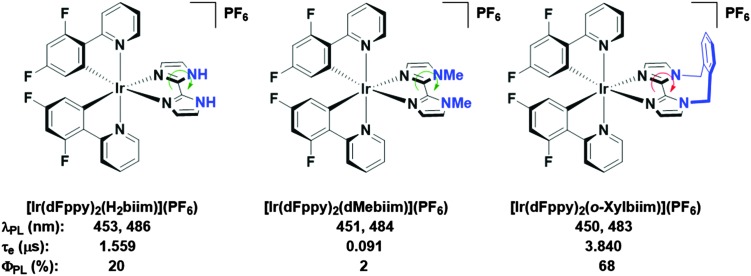
Biimidazole complexes previously studied by our group.^11*b*^ A green arrow connotes free rotation about the C–C bond, while the red arrow connotes the opposite. Solvent: MeOH.

In our own research efforts, we have identified, among other motifs, nitrogen-rich heterocyclic ancillary ligands as promising candidates in this endeavour.^11^ In particular, we have shown biimidazole-type N^∧^N ancillary ligands to be effective motifs for invoking significant destabilisation of the LUMO energies of these complexes (Fig. 1). Crucially, using these scaffolds we demonstrated that very bright blue emission could be realised through alkylation of the distal nitrogen atoms with a rigid *o*-xylylene linker.^11*b*^ Efficient non-radiative decay processes observed for the protonated ([Ir(dFppy)_2_(H_2_biim)](PF_6_), where dFppy is 2-(4,6-difluorophenyl)pyridinato and H_2_biim is 1*H*,1′*H*-2,2′-biimidazole) and especially the methylated ([Ir(dFppy)_2_(dMebiim)](PF_6_), where dMebiim is 1,1′-dimethyl-2,2′-biimidazole) analogues could be strongly suppressed in ([Ir(dFppy)_2_(*o*-xylbiim)](PF_6_), where *o*-xylbiim is 1,1′-(α,α′-*o*-xylylene)-2,2′-biimidazole), to give exceptionally bright emission in MeOH solution (*Φ*_PL_ = 68%, *λ*_em_ = 450 nm). However, poor solubility of these complexes prevented them from being used in solution-processed devices.

**Fig. 2:**
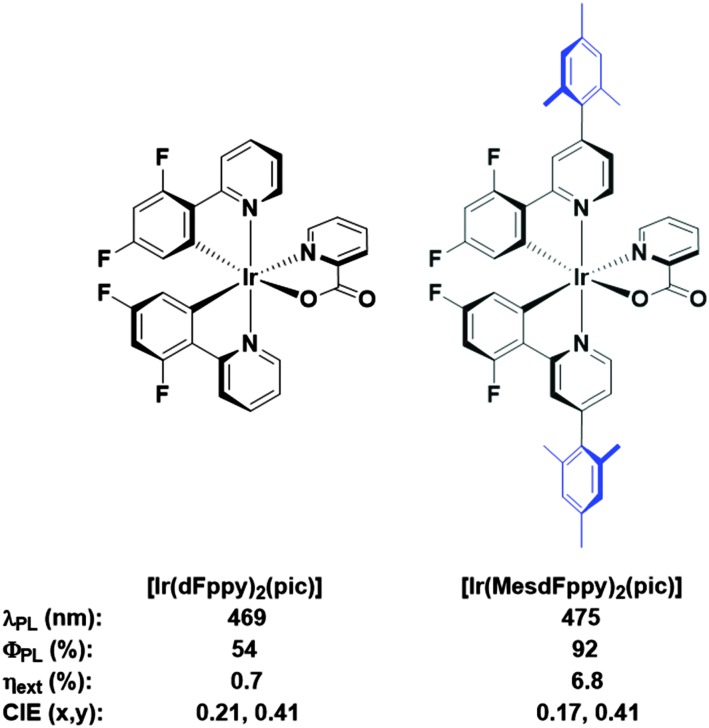
FIrpic and its mesityl functionalised analogue. The mesityl functionality confers improved properties in virtually all important metrics, including solution state *Φ*_PL_, as well as *η*_ext_ values and CIE coordinates for comparable solution processed device architectures. This comes only at the expense of a small red-shift in solution state *λ*_max_.

We had previously demonstrated that aryl-substitution of the diimine ligand could dramatically improve solution-state photoluminescence quantum efficiency, leading to improved stability in the electroluminescent device.^12^ In further surveying the literature for routes towards soluble analogues, we build on the work of Bryce and coworkers, in which they demonstrate vastly improved solution-processed OLED performances using a mesityl-functionalised FIrpic-type emitter (Fig. 2, FIrpic = bis[2-(4,6-difluorophenyl)pyridinato-*C* ^2^,*N*](picolinato)iridium(iii)).^6*b*^ Crucially, the mesityl group accomplishes a number of roles: (1) improved solubility to allow formation of high quality spin-coated thin films from solution; (2) increased steric bulk for inhibiting intramoleculer quenching processes, giving much brighter emission both in solution and in the device; (3) mutual orthogonality of the mesityl group with respect to the cyclometalating ligand, which truncates any extension of the π-conjugation which would invoke an unwanted red-shift in emission.

**Chart 1:**
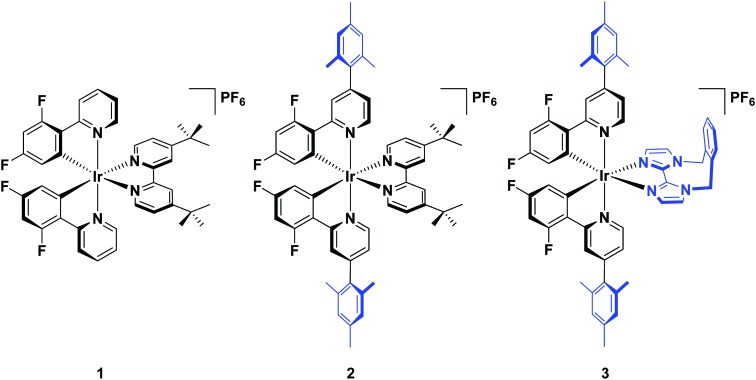
Complexes synthesised and characterised in this study.

Herein, we report a highly soluble, very bright blue biimidazole analogue, [Ir(dFMesppy)_2_(*o*-xylbiim)](PF_6_) (**3**), which can be used in solution-processed OLEDs and LEECs (Chart 1). A previously studied green-emitting iridium complex, [Ir(dFppy)_2_(dtBubpy)](PF_6_) (**1**) serves as the ref. 13 while its mesityl analogue, [Ir(dFMesppy)_2_(dtBubpy)](PF_6_) (**2**) is included to probe the effect of mesityl substitution on the optoelectronic properties of the complex. We study the photophysical properties of these three complexes in acetonitrile solution, and also the photophysics of **3** in methanol to compare to [Ir(dFppy)_2_(*o*-xylbiim)](PF_6_) (**4**). OLEDs have been fabricated from complexes **1–3**. To date, very few examples of OLEDs have been reported employing charged iridium complexes.^14^

## Results and discussion

### Synthesis

**Scheme 1:**
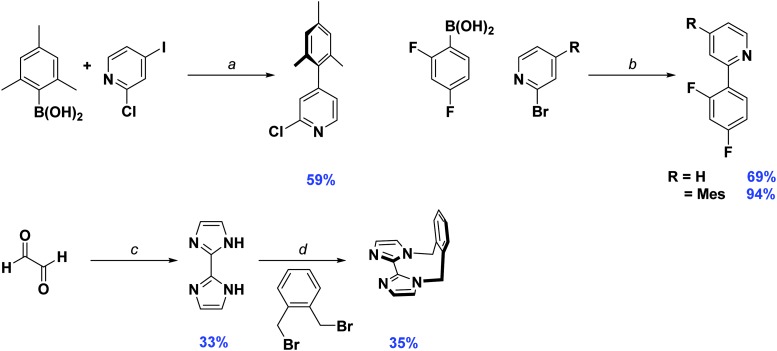
Synthesis of C^∧^N and N^∧^N ligands. *Reagents and conditions*: ^*a*^ K_2_CO_3_, 1,4-dioxane/water (2 : 1 v/v), Pd(PPh_3_)_4_ (5 mol%), 100 °C, 72 h. ^*b*^ Na_2_CO_3_, 1,4-dioxane/water (2 : 1 v/v), Pd(PPh_3_)_4_ (5 mol%), 100 °C, 19 h. ^*c*^ NH_4_OAc, H_2_O, 40 °C, 8 h. ^*d*^ MeCN, NaOH (35% w/v), 82 °C, 16 h.

Synthesis of the cyclometalating ligand, dFMesppy, proceeded through a modified procedure to that reported previously.^6*b*^ The reported method employed sequential Suzuki–Miyaura^15^ cross coupling reactions starting from 2-chloro-4-iodopyridine with first mesitylboronic acid and 2,4-difluorophenylboronic acid as the corresponding coupling partners. While the second step is straightforward, isolation of the 2-chloro-4-mesitylpyridine intermediate after the first step is more problematic. The original procedure stipulated carrying out the reaction in a 1 : 1 stoichiometric ratio of mesitylboronic acid and 2-chloro-4-iodopyridine, presumably to avoid secondary cross-coupling at the 2-position of the pyridine. However, we found that mesitylboronic acid is prone to deborylation as a competitive undesired side reaction under these conditions due to the bulk of the methyl groups in the 2,6-positions. The starting pyridine was thus always recovered from the reaction when carried out in this manner. Since the starting material has virtually the same *R*_f_ as the product, isolating the desired precursor is not possible by column chromatography alone, requiring an additional Kugelrohr distillation step to obtain the product in good purity. To facilitate the purification process, we found that adding a large excess of boronic acid (1.5–1.8 equivalents) leads to complete consumption of the starting material. Any side products generated from over cross coupling under our conditions were separable by column chromatography. Aside from dFMesppy, all other ligands were synthesised in a similar manner to that reported previously;^11*b*^ dFppy was obtained by conventional Suzuki–Miyaura conditions, while the synthesis of *o*-xylbiim proceeded first by a condensation reaction with glyoxal in the presence of ammonium acetate to give H_2_biim, followed by alkylation with 1,2-bis(bromomethyl)benzene (Scheme 1).

**Scheme 2:**
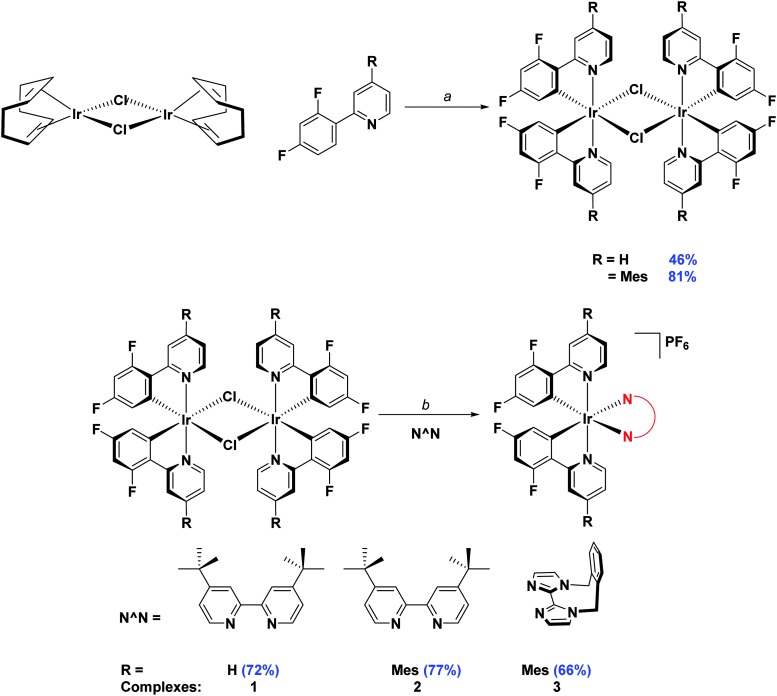
Synthesis of **1–3**. *Reagents and conditions*: ^*a*^ 2-EtOC_2_H_4_OH, 110 °C, N_2_, 3 h. ^*b*^ (i) CH_2_Cl_2_/MeOH (1 : 1 v/v), 50 °C, 19 h, N_2_; (ii) Excess NH_4_PF_6_(aq).

Initially, the complexes were synthesized through cleavage of the dichloro-bridged iridium dimer [Ir(C^∧^N)_2_(μ-Cl)]_2_ with a small excess of ancillary ligand in refluxing DCM/MeOH solution. However, we found that following Nonoyama's method,^16^ in which the precursor dimer complex is obtained from IrCl_3_·3H_2_O and a small excess of cyclometalating ligand, meant that we were never able to isolate our desired complexes in good purity. Instead we found that using [Ir(COD)(μ-Cl)]_2_ (where COD is 1,5-cyclooctadiene) as the initial iridium source gave vastly improved results.^17^ Complexation conditions are summarised in Scheme 2.

### Characterisation

**Fig. 3:**
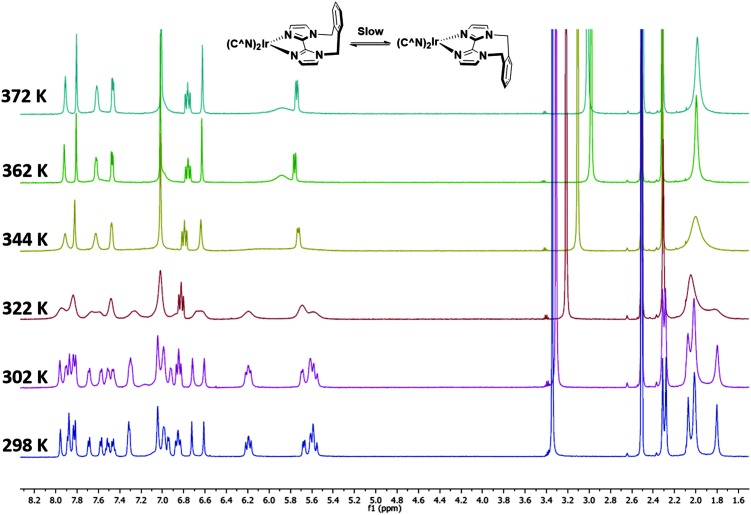
^1^H NMR temperature study of **3** in DMSO-*d*_6_ from room 298 K to 372 K.

Complexes **1–3** were characterised by NMR spectroscopy (^1^H, ^13^C, ^19^F), high resolution mass spectrometry and elemental analysis. As with [Ir(dFppy)_2_(*o*-xylbiim)](PF_6_),^11*b*^ the ^1^H NMR spectrum of **3** presents itself as a complex mixture of broad signals. The complexity of this spectrum comes from the conformational rigidity of the *o*-xylbiim ligand, which means the fluxional ring flipping processes are slow on the NMR time scale at room temperature. Since the iridium centre is itself chiral, coordination to this ligand results in diastereomeric atropisomers. Similarly, the loss of pseudo-*C*_2_ symmetry on the NMR timescale leads to a ^19^F spectrum in which each fluorine atoms is in its own unique magnetic environment, with the spectrum presenting three peaks, two of which corresponding to one fluorine atom and another broad multiplet integrating to double the intensity of the other two peaks (see the ESI†). Heating the solution to 80 °C (372 K) resolved the ^1^H spectrum to one exhibiting the expected *C*_2_-symmetry (Fig. 3). Eyring analysis on the coalescing doublet at 6.7 ppm at 49 °C (322 K) gave a Gibbs free energy barrier to inversion of +72.29 kJ mol^–1^ similar to the previously reported value for [Ir(dFppy)_2_(*o*-xylbiim)](PF_6_) (+82.97 kJ mol^–1^).^11*b*^

### X-ray structure analysis

**Fig. 4:**
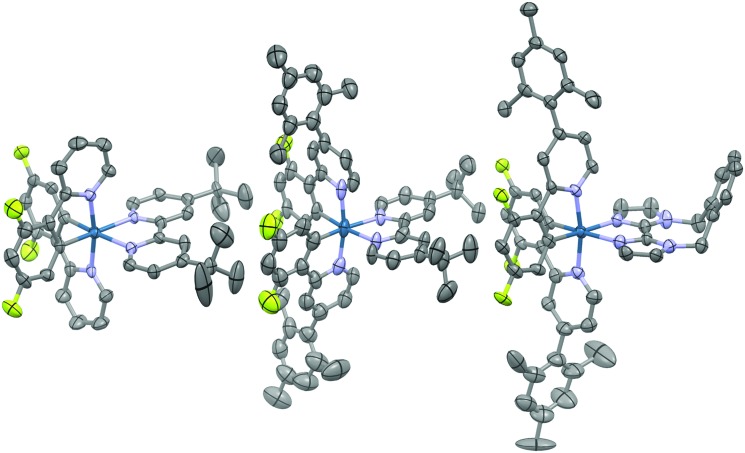
X-ray crystal structures of complexes **1–3**. Hydrogen atoms, solvent molecules and counterions have been removed for clarity.

Suitable single crystals for X-ray analysis were obtained for all three complexes by vapour diffusion of diethyl ether (**1** and **3**) or diisopropyl ether (**2**) into concentrated acetonitrile solutions (Fig. 4). Surprisingly, no single crystal data for **1** has been reported previously. Analysis of crystal packing can be useful for providing an insight into how the molecules might preferentially be arranged within the film. Although the spin-coating process is intended to deposit amorphous films, intermediate-range ordering within spin-coated films of [Ru(bpy)_3_](PF_6_)_2_ has nonetheless been observed,^18^ with such crystallinity suggested to impact the performance of the corresponding electroluminescent devices.^19^

All three complexes display the expected pseudo-octahedral geometry, with the nitrogen atoms of the C^∧^N ligands oriented in a *trans* disposition. Bond lengths and bond angles are also as expected for this class of iridium complex. The crucial design feature that is the large torsion of the mesityl ring with respect to the phenylpyridine moiety is observed for complexes **2** and **3**, with dihedral angles ranging from 69.2(12)°–88.6(19)°.

**Fig. 5:**
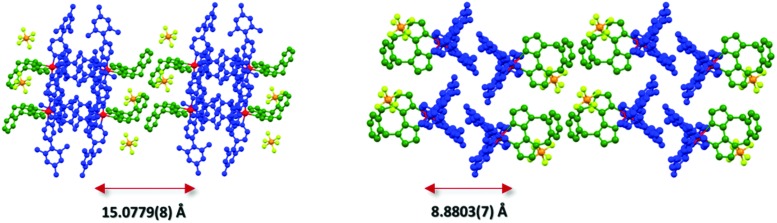
X-ray packing of **3** crystal viewed along the plane of the N^∧^N ligand (left) and from above the cyclometalating ligand N–Ir–N axes (right). For clarity, the iridium atoms have been coloured red, the cyclometalating C^∧^N ligands blue, the N^∧^N ligands green and the PF_6_^–^ counterions yellow (fluorine) and orange (phosphorous). Hydrogens and solvent molecules are omitted. The distance on the left reflects the iridium–iridium distance across the N^∧^N ligands, while the distance on the right reflects the iridium–iridium distance across the C^∧^N ligands.

An analysis of the extended crystal packing reveals features that explain several photophysical phenomena observed in the solid state (*vide infra*). All three complexes show a similar shortest Ir···Ir internuclear distance [**1**: 8.5148(9) Å, **2**: 9.000(3) Å, **3**: 8.8803(7) Å], despite the other differences observed in intermolecular interactions. Complex **1** shows no strong intermolecular interactions in the solid state, with only weak C–H···π hydrogen bonds observed. In contrast, despite the added bulk resulting from the mesityl groups present in **2** and **3**, these complexes show additional intermolecular interactions, with complexes positioned such that two difluorophenyl rings of adjacent complexes are correctly positioned to make a π-stacking interaction, with centroid···centroid distances of 3.628(8) and 3.531(7) Å, respectively, facilitated by the propensity of fluorinated phenyl rings to more readily form π-stacking interactions than fluorine-free rings.^20^ These interactions are strengthened by further mutual C–H···π contacts between the same adjacent complexes. The packing in **3** is further ordered through a secondary π-stacking interaction between the xylyl rings of the *o*-xylbiim ligand of adjacent complexes. This combination of π-interactions results in the formation of strongly interacting chains of molecules running along the crystallographic *ab*-diagonal axis, with alternating long [15.0779(8) Å] (Fig. 5, left) and short [8.8803(7) Å] iridium–iridium distances (Fig. 5, right).

### Electrochemical properties

**Fig. 6:**
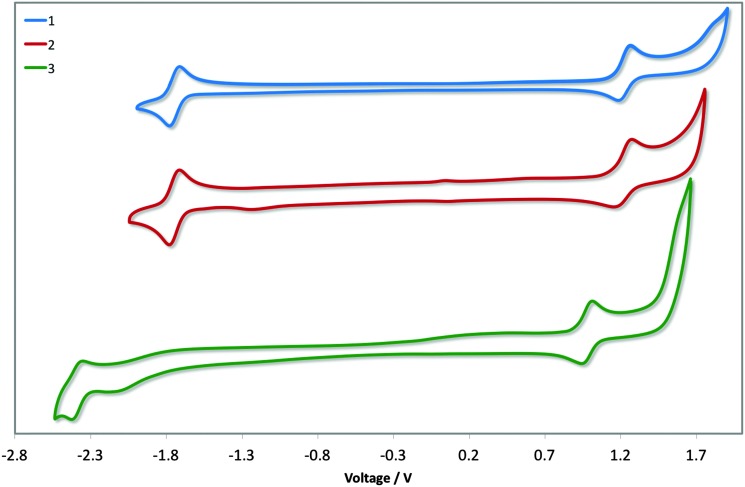
CV traces of complexes **1–3** in MeCN solution, reported *versus* SCE (Fc/Fc^+^ = 0.38 V in MeCN)^22^ redox couple. Scan rates were at 100 mV s^–1^, and are in the positive scan direction.

Cyclic voltammetry was undertaken to discern the energy levels of the complexes, which is crucial for device fabrication purposes. The CV traces of **1–3** in acetonitrile are given in Fig. 6, and the relevant electrochemical parameters obtained for these complexes are summarised in Table 1. Electrochemical data in dichloromethane had previously been reported for **1**. The previously reported values (*E*ox1/2: 1.17 V; *E*red1/2: –1.80 V *vs.* SCE; Δ*E*_redox_: 2.97 V)^13*b*^ differ marginally from our own (*E*ox1/2: 1.22 V; *E*red1/2: –1.74 V *vs.* SCE; Δ*E*_redox_: 2.96 V); the modest shifting to more positive potential in MeCN is due to its increased polarity, which confers a greater stabilization of both the HOMO and LUMO. DFT calculations for **1** have shown the electron density of the HOMO to be localised on the metal centre and on the C^∧^N ligands while the LUMO is largely localised on the N^∧^N ancillary, distributions characteristic of cationic iridium complexes.^21^ Thus, we assign the HOMO for all three complexes to the Ir^III^/Ir^IV^ redox couple, with contributions from the phenyl components of the C^∧^N ligands, while the reduction process occurs on the dtBubpy in the case of **1** and **2**. Indeed, the introduction of the mesityl groups in **2** have a negligible effect on its electrochemistry compared to **1**.

**Table 1:** Relevant electrochemical data for complexes **1–3** ^*a*^

Complex	*E* ox 1/2 (V)	*E* red 1/2 (V)	Δ*E* (V)	*E*_HOMO_ ^*b*^ (eV)	*E*_LUMO_ ^*b*^ (eV)
**1**	1.60	–1.36	2.96	–6.03	–3.06
**2**	1.59	–1.36	2.95	–6.01	–3.06
**3**	1.37	–1.99	3.36	–5.79	–2.43

^*a*^Measurements were carried out in MeCN at a scan rate of 100 mV s^–1^ with Fc/Fc^+^ employed as an internal standard, and reported *vs.* SCE (Fc/Fc^+^ = 0.38 V in MeCN).^22^ Reported potentials are referenced to the Fc/Fc^+^ redox couple.

^*b*^ *E*_HOMO/LUMO_ = –[*E* ^ox/red^ *vs.* Fc/Fc^+^ + 4.8] eV.^23^

The electrochemical properties of **3** differ markedly from **1** and **2**. Both the oxidation and reduction potentials of **3** are shifted cathodically compared to **1** and **2**. The reduction wave in **3** shows much poorer reversibility compared to the corresponding reduction waves of **1** and **2**. The destabilisation of the HOMO of **3** (5.79 eV) compared with **1** and **2** (*ca.* –6.0 eV) is offset by the significant destabilisation of the LUMO (–2.37 eV *vs.* –1.74 eV for **1** and **2**) resulting in a much larger electrochemical gap of 3.36 V. Although the oxidation process in **3** is not likely to involve the *o*-xylbiim ligand directly, its strong electron-releasing potential nevertheless strongly influences the largely metal-localised HOMO. The electrochemical data for [Ir(dFppy)_2_(*o*-xylbiim)](PF_6_), **4**, in MeCN demonstrated an oxidation at 1.06 V, although a reduction process was not observed within the solvent window for that complex.^11*b*^

### UV-vis absorption

**Fig. 7:**
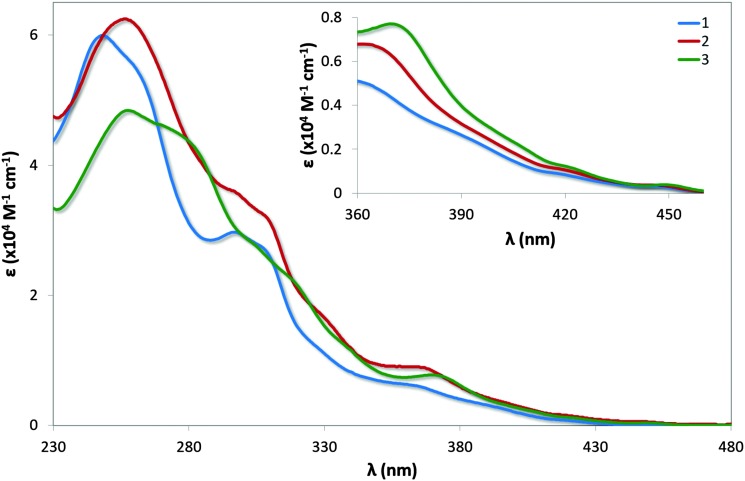
UV-vis absorption spectra of complexes **1–3** in MeCN solution. Inset: zoomed region of absorption spectra.

UV-vis absorption spectra for **1–3** are shown in Fig. 7 with the data summarised in Table 2. The profile observed for **1** generally reproduces that reported in the literature, with the principal band at 249 nm and a shoulder at 298 nm assigned as typical π–π* transitions associated with this family of complexes. The bands at energies lower than 360 nm are assigned as a combination of singlet and triplet metal-to-ligand and ligand-to-ligand charge transfer (^1^MLCT/^1^LLCT and ^3^MLCT/^3^LLCT) transitions.^24^ The mesityl groups in **2** confer only a small change in the profile of the spectrum with increased molar absorptivities across the spectrum; the shoulder at 261 nm present in **1** is not observed in **2**.

**Table 2:** Absorption maxima and their corresponding molar absorptivities for complexes **1–3** ^*a*^

Complex	*λ*_abs_ (nm) [*ε* (×10^4^ M^–1^ cm^–1^)]
**1**	249 [6.00], 261(sh) [5.46], 298 [3.02], 307(sh) [2.74], 365 [0.48], 420 [0.08], 450 [0.04]
**2**	257 [6.25], 297(sh) [3.60], 309(sh) [3.21], 330(sh) [1.66], 368 [0.65], 420 [0.11], 449 [0.04]
**3**	258 [4.84], 281(sh) [4.33], 306(sh) [2.73], 321(sh) [2.13], 371 [0.77], 422 [0.12], 450 [0.04]

^*a*^Measurements were carried out in MeCN.

Similar to that observed in **2**, the presence of the mesityl substituents in **3** have minimal impact on the absorption profile with only modest increases in the molar absorptivities compared to [Ir(dFppy)_2_(*o*-xylbiim)](PF_6_), **4**. The shoulder at 281 nm for **3** is considerably more pronounced than the shoulder at 279 nm for **4**, which is likely the result of π–π* transitions localised on the mesityl ring. Although the measurements for **4** were carried out in MeOH while those for **3** are reported in MeCN, the highly ligand-centred (^1^LC) nature of the excited state of these complexes results in negligible solvatochromic effects.

### Emission spectroscopy

**Fig. 8:**
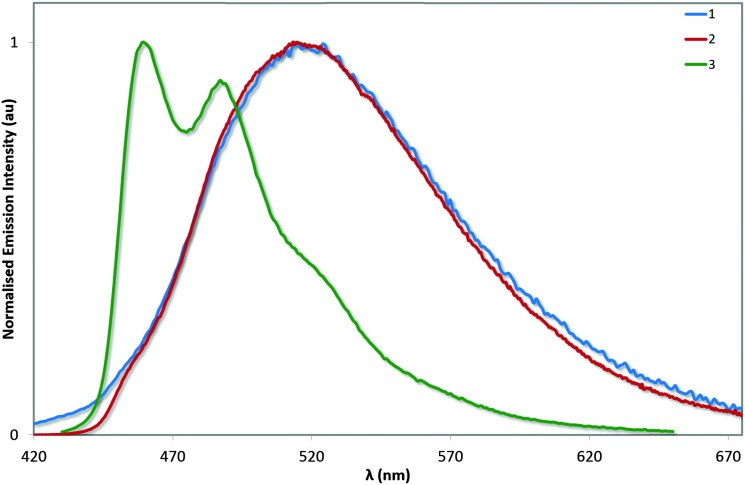
Normalised emission spectra for complexes **1–3** in deaerated MeCN solution. *λ*_exc_: 360 nm.

The photoluminescence properties of these complexes were studied in MeCN solution and are collected in Table 3 and Fig. 8. On the basis of the microsecond lifetimes observed for all three complexes (*τ*_e_ > 1.3 μs), the emission is ascribed as phosphorescence, as is typical of many cyclometalated iridium(iii) complexes. In order to render a more accurate comparison with **4**, the photophysics of **3** was also studied in MeOH. The photophysical behaviour measured for **1** generally reproduced that described previously (avg. *Φ*_PL_ = 70–71%; *τ*_e_ = 1.25–1.40 μs), although two different *λ*_max_ values have been reported previously (512 and 524 nm):^13^ we observe bright green emission (*λ*_em_ = 515 nm; *Φ*_PL_ = 72%; *τ*_e_ = 1.36 μs) from a mixed ^3^MLCT/^3^LLCT state from our own sample, as evidenced by the broad unstructured emission profile.

**Table 3:** Relevant solution state photophysical data for complexes **1–3** ^*a*^

Complex	*λ*_em_ ^*b*^ (nm)	*Φ*_PL_ ^*c*^ (%)	*τ*_e_ ^*d*^ (μs)	*k*_r_ × 10^5^ s^–1^	*k*_nr_ × 10^5^ s^–1^
**1**	515	72	1.36	5.29	2.06
**2**	515	80	1.37	5.84	1.46
**3**	459, 487	90	2.19	4.11	0.46
**3** ^*e*^	458, 489	82	2.26	3.63	0.80
**4** ^*e*^ ^,^ ^*f*^	457, 487	68	3.84	1.77	0.83

^*a*^Measurements at 298 K in deaerated MeCN unless otherwise stated otherwise.

^*b*^ *λ*_exc_: 360 nm.

^*c*^Quinine sulfate used as the reference (*Φ*_PL_ = 54.6% in 0.5 M H_2_SO_4_ at 298 K).^25^

^*d*^ *λ*_exc_: 375 nm.

^*e*^Measured at 298 K in deaerated MeOH.

^*f*^Data taken from ref. 11*b*.

The electronics of the system remain unchanged between **1** and **2**, analogous to that observed between FIrpic and its mesityl-functionalised analogue.^6*b*^ This observation is consistent with the absorption spectroscopy and the electrochemistry. However, the excited state kinetics are noticeably different. The *Φ*_PL_ observed for **2** is higher than for **1**, despite exhibiting virtually the same *τ*_e_; the result of a combination of slightly increased *k*_r_ and decreased *k*_nr_. By comparison, the study of **3** and **4** in MeOH show this increased brightness (*Φ*_PL_ = 82% for **3** *vs.* 68% for **4**) is almost entirely due to an enhancement of *k*_r_ resulting from the reduced triplet–triplet quenching promoted by the bulky mesityl groups. Complex **3** is extremely bright in MeCN solution with a *Φ*_PL_ of 90%. The improved brightness of **3** compared with **1** and **2** is likely to be due to a combination of energy gap law effects arising from the strongly blue-shifted emission of **3** as well as the strongly rigidifying effects of the *o*-xylbiim ligand. Ultimately, we have demonstrated that a blue emitter with near unitary *Φ*_PL_ is achievable through the combination of mesityl substituents on the C^∧^N ligands (which inhibit intermolecular quenching) and the use of the *o*-xylbiim ancillary ligand (which strongly inhibits intramolecular non-radiative processes).

To the best of our knowledge this is the highest photoluminescence quantum yield for a blue cationic complex reported to date; all cationic iridium emitters that have a bluer *λ*_max_ are significantly less efficient. While this is no doubt in part due to more thermally accessible MC states in these emitters as a function of a larger HOMO–LUMO gap, this result nevertheless demonstrates the potency of our ligand design. For example, Baranoff *et al.* reported a deep blue-emitting iridium complex (440 nm in MeCN) based on a bis-NHC ancillary ligand, but this complex has a *Φ*_PL_ of only 13%.^26^ Similarly, we recently reported a series of complexes bearing multiple 1,2,3-triazole heterocycles, and these too demonstrated moderately bluer emission (452 nm for the best example) but a much lower *Φ*_PL_ of 5%.^27^ On the other hand, the most efficient example among these 1,2,3-triazole complexes was also the least blue (487 nm, *Φ*_PL_ = 30%) and still lower in *Φ*_PL_ than **3**. The group of Qiu *et al.* have focussed on incorporating pyrazole and imidazole heterocycles into their ligand scaffolds with a view to blue-shifting the emission of their complexes. Although they have achieved significant results, none of their complexes have been reported to be more efficient than **3** (highest *Φ*_PL_ of 54%), while their bluest is only moderately bluer than our own (*λ*_max_ = 456 nm).^7,27,28^

**Fig. 9:**
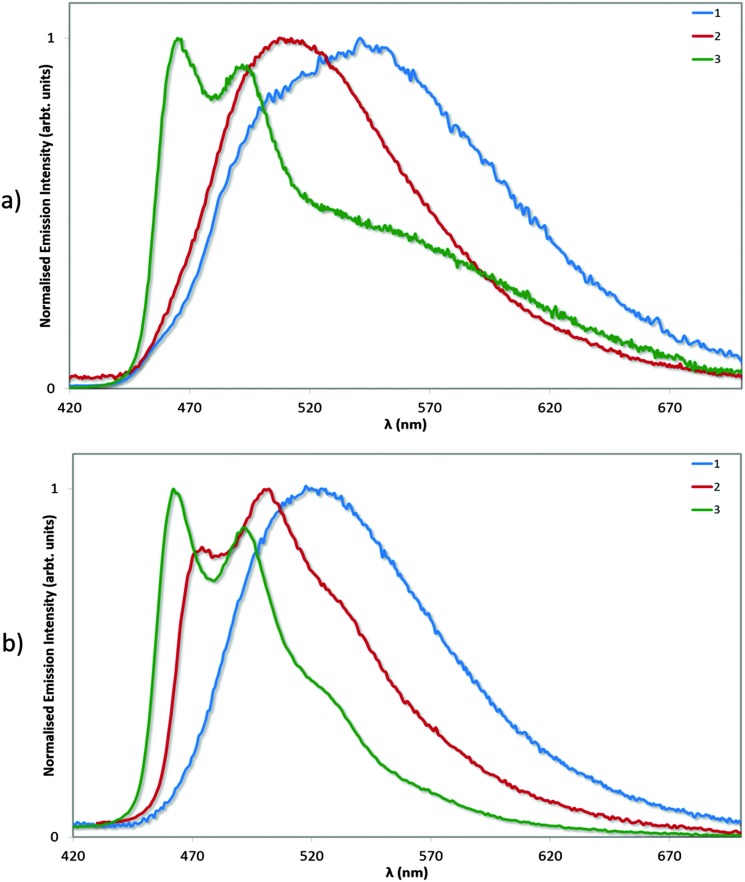
Normalised emission spectra for neat (a) and doped (5 wt% in PMMA, b) films of complexes **1–3**. *λ*_exc_: 360 nm.

Photophysical measurements in the solid state were also performed with a view to correlating these measurements to the crystal packing observations made above. Measurements were carried out both in neat and doped (5 wt% in PMMA) films, and the results collated in Table 4 and the spectra shown in Fig. 9. We find that the *Φ*_PL_ values decrease across the series from **1** (62%) to **2** (54%) to **3** (43%). The decrease in *Φ*_PL_ may be linked to the propensity of **2** and particularly **3** to form more ordered crystal packing arrays than **1**, which may make these complexes more susceptible to concentration quenching processes by means of aggregate formation.^29^ By contrast, the photoluminescence quantum yields of the doped films, as expected, are independent of crystal packing effects, with exceptionally high values for all three complexes. The *Φ*_PL_ for **1** was previously reported to be 96%,^13*a*^ while by comparison we measured 90% following excitation at 300 nm. Under identical conditions, the *Φ*_PL_ of **2** is essentially unitary at 97%! Finally, complex **3** is virtually as bright in the solid state (*Φ*_PL_: 89%) as in MeCN solution (*Φ*_PL_: 90%), indicating that the rigidifying effects in the solid state are limited for this complex since this has already largely been achieved by the *o*-xylbiim ligand. These are remarkably high *Φ*_PL_ values for a charged iridium complex in the solid state.

**Table 4:** Relevant solid-state photophysical data for complexes **1–3**

Complex^*a*^	*λ*_em_ ^*b*^ (nm)	*Φ*_PL_ ^*c*^ (%)	*τ*_e_ ^*d*^ (μs)
**1** (neat film)	520	62	0.41 (41%), 0.85 (59%)
**1** (doped film)	518	90	1.75
**2** (neat film)	508	54	0.39 (68%), 1.23 (34%)
**2** (doped film)	474, 502	97	1.63
**3** (neat film)	465, 492	43	0.19 (29%), 1.23 (71%)
**3** (doped film)	462, 492	89	1.92

^*a*^Neat films were dip coated from MeCN solution while doped films were dip coated from a DCM solution of 5 wt% of the complex in PMMA.

^*b*^ *λ*_exc_ at 360 nm.

^*c*^Measured using an integrating sphere, with *λ*_exc_ for the neat film performed at 360 nm and for the doped films at 300 nm.

^*d*^ *λ*_exc_ at 378 nm.

We have extensively analysed reported *Φ*_PL_ values for ionic iridium(iii) emitters in the solid state, and found that very few exceed the values we report here. We note that although complexes **2** and **3** show significantly diminished *Φ*_PL_ in neat film compared to solution or doped films, these values still compare well with both ‘classic’ iridium complexes, which do not possess large, bulky substituents, such as *fac*-Ir(ppy)_3_ (*Φ*_PL_ = 97% in a 1.5 mol% CBP film compared to *Φ*_PL_ = 3% in neat film)^30^ and FIrpic (*Φ*_PL_ = 99% in 1.4 mol% mCP film compared to *Φ*_PL_ = 15% in neat film),^30^ as well as with other iridium complexes bearing bulky ligand functionalities such as Wong's 4,5-diaza-9,9′-spirobifluorene (*Φ*_PL_ = 67% in a 1.5 mol% mCP film compared to *Φ*_PL_ = 32% in neat film).^14*d*^

In doped film, aside from the *Φ*_PL_ of 96% reported previously for **1**,^13*a*^ only nine cationic complexes have been reported previously with *Φ*_PL_ of similar magnitude. For instance, a series of emitters based on dFppz and varying N^∧^N ligands were reported by Baranoff and co-workers with three within the study showing *Φ*_PL_ ranging from 89% to 100%, but with greener emission than **3** (*λ*_max_ = 500–510 nm).^31^ Tordera and co-workers in the same year reported fluorinated green emitters with similar photophysical behaviour (*Φ*_PL_ ranging from 82% to 93%) at similar wavelengths (503–519 nm).^32^ Housecroft *et al.* reported a number of complexes characterised by cyclometalating ppz ligands decorated with electron-withdrawing sulfone groups on the phenyl moiety and found that they were all near-quantitative emitters in the solid state, with *Φ*_PL_ ranging from 86–94% and *λ*_max_ 487–505 nm.^33^ Qiu's complex bearing a tritylphenyl functionalised phenylimidazole ancillary ligand is the bluest (*λ*_max_ = 474 nm) complex amongst those we could find with reported solid-state emission data, with a high *Φ*_PL_ of 79%;^34^ it is nevertheless less bright and more red-shifted in emission than **3**. It should be noted that all the values reported for these complexes were from samples in doped PMMA films (5 wt%) similar to our own. Of particular note, however, is the blue emitting (*λ*_max_ = 474 nm) complex [Ir(dFppz)_2_(dtbubpy)](PF_6_), which demonstrates a brighter *Φ*_PL_ (75%) in neat film than any of **1–3**.^35^

### Device characterization

#### Electroluminescent devices: organic light-emitting diodes (OLEDs)

**Fig. 10:**
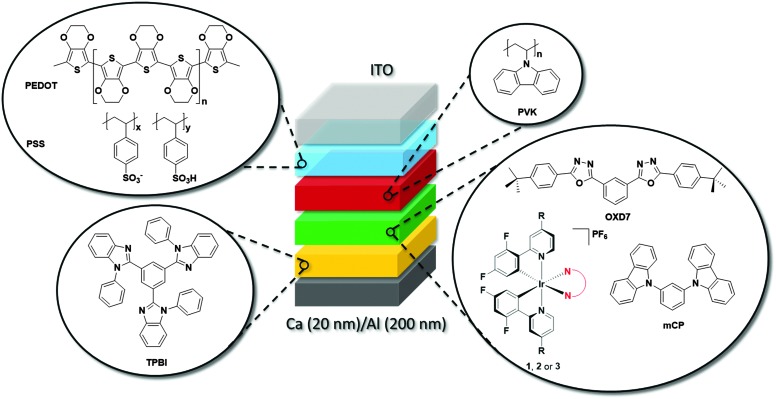
Schematic for OLED device. The architecture consisted of the following structure: ITO/PEDOT:PSS (40 nm)/PVK (35 nm)/(mCP + active layer + OXD7) (30 nm)/TPBI (60 nm)/Ca (20 nm)/Al (200 nm) structure, where **1**, **2** or **3** act as the active layer.

In view of their promising thin film photophysical properties **1**, **2** and **3** were investigated as emitters in OLEDs (Fig. 10). The emitter was embedded in an OXD7/mCP host, and sandwiched between organic layers of PVK and TPBI. PVK facilitates the injection of holes, while the electron transport layer TPBI blocks the holes from penetration into the cathode due to a deep lying HOMO, reducing current leakage. Such a multi-layer structure helps to confine the excitons within the emitting layer as is needed for good OLED performance. Except for TPBI and the contacts, all the layers were deposited by solution-processing methods. Device performance is summarized in Table 5.

**Table 5:** OLED performance data

Complex	Turn-on voltage (V)	Luminance (cd m^–2^) max	EQE (%)@100 cd m^–2^	EQE (%) max	Power efficiency (lm W^–1^)@100 cd m^–2^	Power efficiency (lm W^–1^) max	CIE Coordinates (*x*, *y*)
**1**	6.4	1790	3.74	5.42	2.92	6.23	0.25, 0.48
**2**	6.2	2940	3.12	3.95	2.62	4.81	0.21, 0.40
**3**	4.8	1090	2.86	3.42	2.42	4.42	0.21, 0.37

**Fig. 11:**
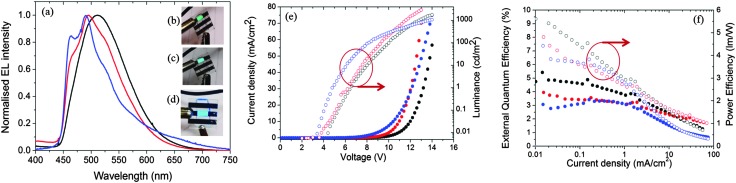
(a) Normalized electroluminescent emission spectra of **1** (black), **2** (red) and **3** (blue) in thin films. Photograph of the working OLED with (b) **1** (c) **2** and (d) **3**. (e) Current density and luminance *versus* applied voltage for OLEDs. (f) External quantum efficiency (solid circles) and power efficiency (open circles) *versus* applied voltage for OLEDs made with **1** (black) and **2** (red) and **3** (blue).

Fig. 11a shows the electroluminescence spectra of the three devices. The spectra are broad for **1**, modestly structured for **2**, and structured for **3**. The EL spectra exhibit similar profiles to the film photoluminescence spectra, except for the change in the relative intensity of the vibronic peaks for **3** and a small blue shift for **1** and **2**. The photographs for actual working OLEDs for **1**, **2** and **3** are shown in Fig. 11b, c and d, respectively. The Commission International de L'Éclairage (CIE) colour coordinates of the OLED using complex **1** are (0.25, 0.48). As observed with the neat PL films, incorporation of the mesityl groups in **2** results in a blue-shift of the CIE coordinates to (0.21, 0.40). The replacement of the d*t*Bubpy ligand in **2** with the *o*-xylbiim ligand in **3** unfortunately does not lead to substantially bluer CIE coordinates, which were found to be (0.21, 0.37). In the EL spectra, the emission from mCP:OXD-7 (expected around 410 nm) disappears completely indicating that complete energy transfer occurs from mCP:OXD-7 mixed host system to all the emissive complexes.

Fig. 11e shows the current–voltage–luminance characteristics of the three devices. The turn-on voltage is lowest for the device made with **3** as the active layer, which is 4.8 V. The turn-on voltages for other devices are similar at around 6.3 V. The maximum luminescence of 2935 cd m^–2^ is achieved for devices using **2** as the active layer at a driving voltage of 11.8 V. Fig. 11f shows the external quantum efficiency (EQE) of the devices together with the power efficiency. The maximum EQE is achieved for the reference complex **1**. The efficiencies of these devices are reduced at high brightness. This is likely due to the deterioration of charge carrier balance in the device at high current density and the increase of non-radiative quenching processes, including triplet–triplet annihilation (TTA).^36^ These results suggest the performance of the devices can be optimized further by improving the charge balance.

Compared to our devices, the device reported by Bryce demonstrates superior overall efficiency (EQE: 10.4% for the optimised device).^6*b*^ However, we note that charged iridium emitters are a rarity for OLEDs, and it is useful to compare our own devices to what has been reported using emitters of this family. To the best of our knowledge, only a single report exists employing charged iridium complexes as the emitters in a vacuum deposited OLED. The emitters utilised a conjugated diphenylamine–fluorenylpyridine C^∧^N ligand that allowed the inherently poor volatility of cationic iridium complexes to be overcome. These devices show reasonable external quantum efficiencies, peaking at an EQE of 6.5%.^14*d*^ Our solution-processed emitters are not quite as efficient but offer the advantage of simpler processing and the possibility of being patterned by printing processes.

## Conclusions

A series of cationic green- to blue-emitting, fluorinated iridium complexes have been studied. We have shown that both highly efficient and deep blue emission can be achieved in both MeCN solution and in doped PMMA thin films by rational design of the ligand scaffolds that simultaneously suppress inter- and intramolecular quenching pathways. Solution processed OLEDs were fabricated, giving sky blue devices with favorable performances compared with other OLEDs employing charged iridium emitters to date.
