# Supplementary material for: Solubilised bright blue-emitting iridium complexes for solution processed OLEDs
Source: J Mater Chem C Mater. 2016 Feb 11;4(17):3726–37. doi: 10.1039/c6tc00151c (PMC4906369; doi:10.1039/c6tc00151c)
Supplement: Supplementary file 1 [file TC-004-C6TC00151C-s001.pdf]

Electronic Supplementary Material (ESI) for Journal of Materials Chemistry C.
This journal is © The Royal Society of Chemistry 2016

Solubilised Bright Deep Blue-Emitting Cationic Iridium Complexes for Solution Processed OLEDs

*Adam F. Henwood[a], Ashu K. Bansal[b], David B. Cordes[a], Alexandra M. Z. Slawin[a], Ifor D.W. Samuel[b], Eli Zysman-Colman[a]**

[a] Organic Semiconductor Centre, EaStCHEM School of Chemistry, University of St Andrews, St Andrews, Fife, KY16 9ST, UK, Fax: +44-1334 463808; Tel: +44-1334 463826;
[b] Organic Semiconductor Centre, SUPA, School of Physics and Astronomy, University of St Andrews, Fife, KY16 9SS, UK
E-mail: eli.zysman-colman@st-andrews.ac.uk;
URL: http://www.zysman-colman.com

SUPPORTING INFORMATION

Table of contents:

	Pages
Synthesis of precursor compounds	S2-S36
Details of photophysical measurements	S37
X-Ray crystallography	S40
References	S41

2-(2,4-Difluorophenyl)pyridine, (dFppy).

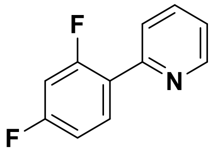

The synthesis of this ligand is as reported previously.[1] 2,4-Difluorophenylboronic acid (1.5 equiv.), 2-bromopyridine (1.0 equiv.), sodium carbonate (2.0 equiv.) were added to a round bottom flask containing a mixture of 1,4-dioxane and distilled water (4:1 v/v) to obtain a concentration of 0.15 to 0.20 M. The reaction mixture was degassed via bubbling nitrogen for 20 minutes. $Pd(PPh_3)_4$ (5 mol%) was quickly added to the round bottom flask, and the reaction mixture was sealed. The mixture was degassed again by evacuating and then backfilling with nitrogen three times, before heating to reflux. The mixture was refluxed for 19 h before cooling to room temperature and pouring onto distilled water. Extraction of the product with multiple portions of ethyl acetate was followed by combining the organic fractions, washing with water and then saturated sodium hydrogen carbonate to remove residual boronic acid. Evaporation under reduced pressure gave the crude product. Purification by flash column chromatography (silica, hexane/ethyl acetate 95:5) gave 0.554 g of pure compound as a colourless oil. **Yield:** 69%. **R_f:** 0.48 (20% EtOAc/hexanes on silica). **^{1}H {^{19}F} NMR (500 MHz, $CDCl_3$) δ (ppm):** 8.72 (dt, *J* = 4.5, 1.0 Hz, 1H), 8.03 - 7.99 (m, 1H), 7.75 (d, *J* = 4.0 Hz, 2H), 7.28 □ 7.23 (m, 1H), 7.03 - 6.99 (m, 1H), 6.93 - 6.90 (m, 1H). **^{13}C NMR (126 MHz, $CDCl_3$) δ (ppm):** 164.6, 162.1, 159.5, 152.7, 149.9, 136.6, 132.3, 124.4, 122.6, 112.1, 104.5. **^{19}F {^{1}H} NMR (471 MHz, $CDCl_3$) δ (ppm):** -109.3 (d, *J* = 6.8 Hz, 1F), -113.0 (d, *J* = 6.8 Hz, 1F). **GCMS:** (13.6 min) $[M]^+$: 191. The characterisation matches that reported.[1]

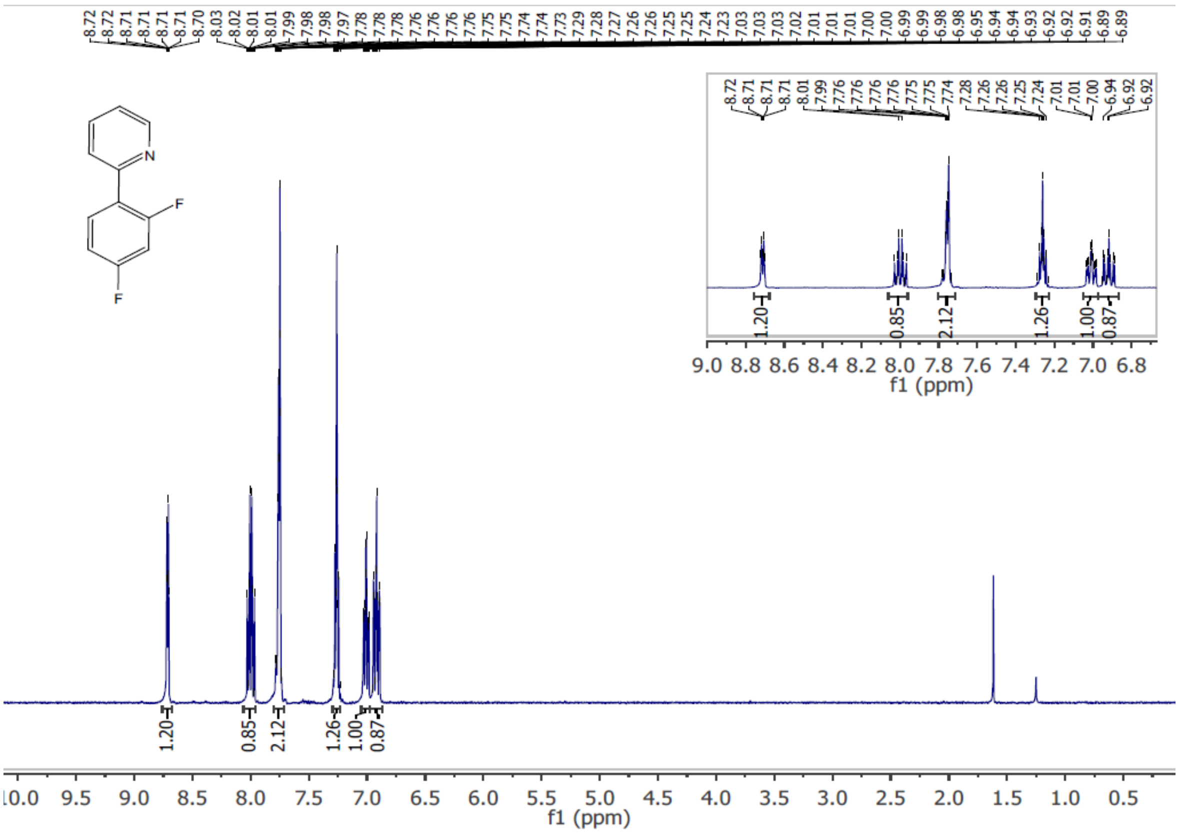

Figure **S1**. ^{1}H NMR spectrum of **2-(2,4-Difluorophenyl)pyridine (dFppy)** in $CDCl_3$.

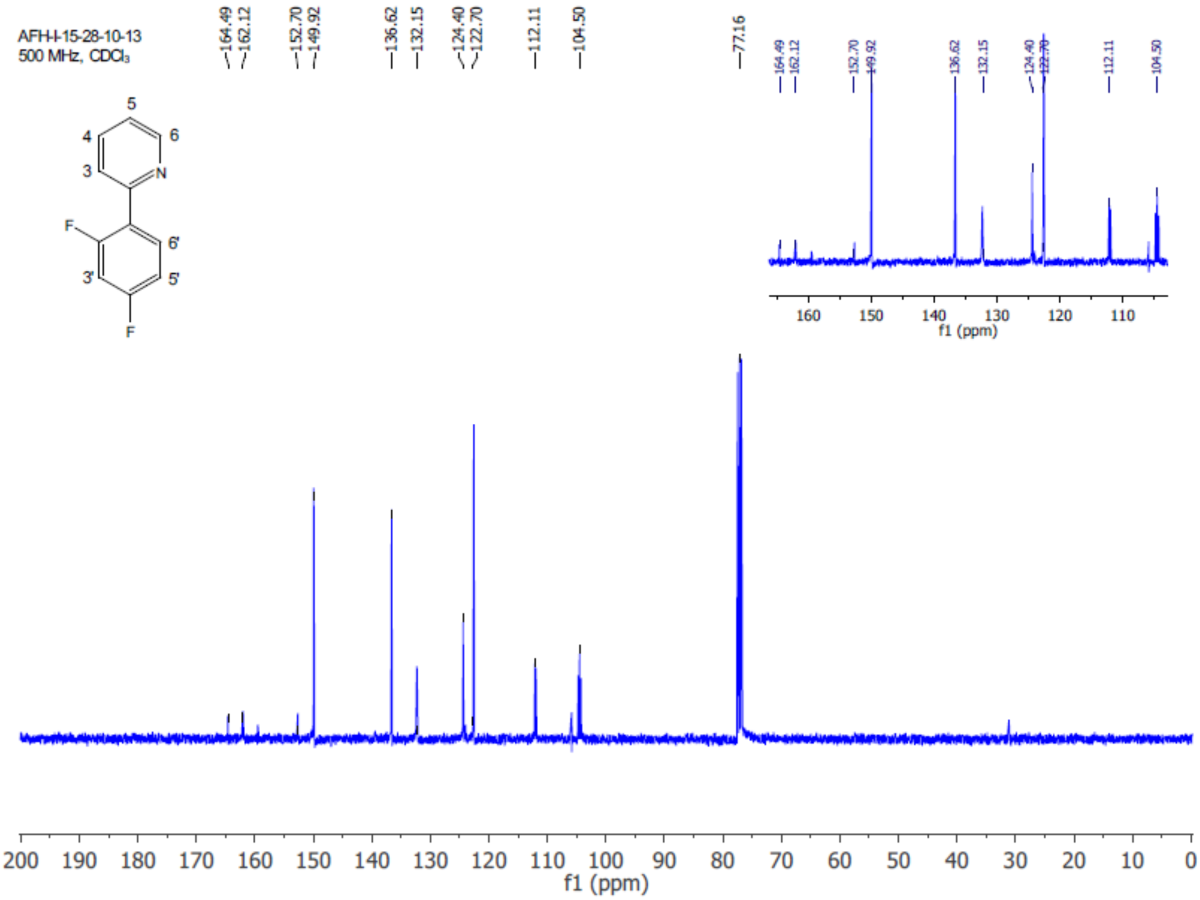

Figure **S2**. ^{13}C NMR spectrum of **2-(2,4-Difluorophenyl)pyridine (dFppy)** in $CDCl_3$.

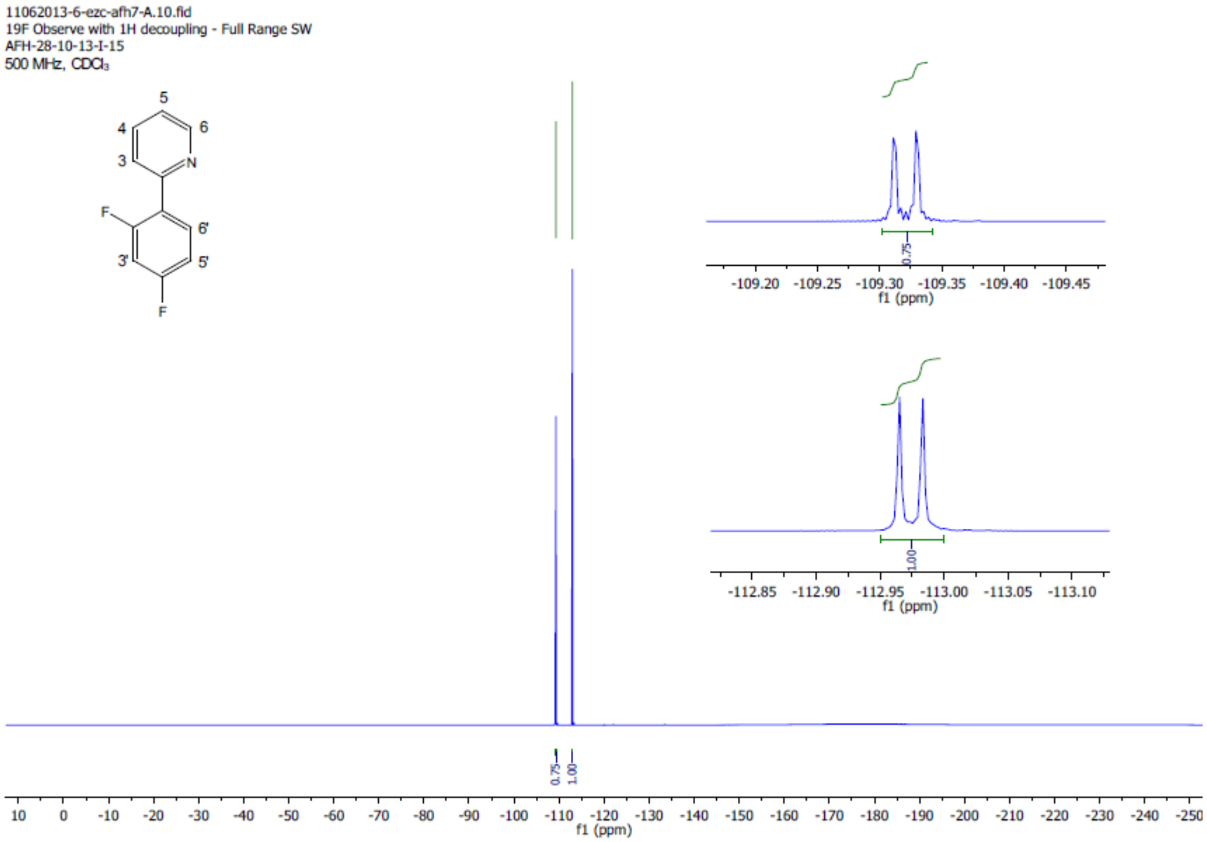

Figure **S3**. ^{19}F {^{1}H} NMR spectrum of **2-(2,4-Difluorophenyl)pyridine (dFppy)** in $CDCl_3$.

2-Chloro-4-(2,4,6-trimethylphenyl)pyridine.

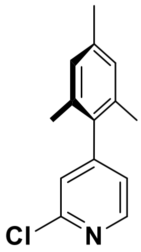

Synthesis of this compound was by a modified procedure to that reported previously.[2] To a round bottom flask was added 2,4,6-trimethylphenylboronic acid (1.7 equiv.), 2-chloro-4-iodopyridine (1.0 equiv.) potassium carbonate (3.0 equiv.), 1,4-dioxane and water (2:1 v/v) to give a solution of ca. 1.25 M. Note: an excess of boronic acid is required to ensure full consumption of 2-chloro-4-iodopyridine, since the boronic acid is prone to deborylation *in situ* and separation of 2-chloro-4-(2,4,6-trimethylphenyl)pyridine from 2-chloro-4-iodopyridine by chromatography is not possible. The reaction mixture was degassed by vigorously bubbling N_2 for 20 min. $Pd(PPh_3)_4$ (5 mol%) was added and the reaction mixture sealed. The vessel was then subjected to three cycles of evacuating under vacuum and backfilling with N_2, before heating the mixture to reflux for 72 h or until the 2-chloro-4-iodopyridine was not visible by NMR. Upon cooling to room temperature, toluene was added and the layers separated. The organic layer was washed with water and brine, and then the solvent was removed *in vacuo* to give the crude material as an oil. Purification by flash column chromatography (silica, hexane, 5% ethyl acetate) gave the pure compound as a colourless oil (2.288 g). **Yield**: 59%. **R_f:** 0.37 (hexane/ethyl acetate, 6:1 on silica). **^{1}H NMR (400 MHz, $CDCl_3$) δ (ppm):** 8.40 (d, J = 4.0 Hz, 1H), 7.12 (s, 1H), 7.02 (dd, J = 1.2, 4.0 Hz, 1H) 6.92 (s, 2H) 2.29 (s, 3H), 1.97 (s, 6H). **^{13}C NMR (126 MHz, $CDCl_3$) δ (ppm):** 152.9, 151.8, 149.8, 138.0, 134.9, 128.5, 125.1, 123.6, 21.0, 20.5. **HR-MS** (APCI$^+$)**: [M+H]$^+$ Calculated:** ($C_{14}H_{14}ClNH$) 234.0858; **Found:** 234.0856. The characterisation matches that reported.[2]

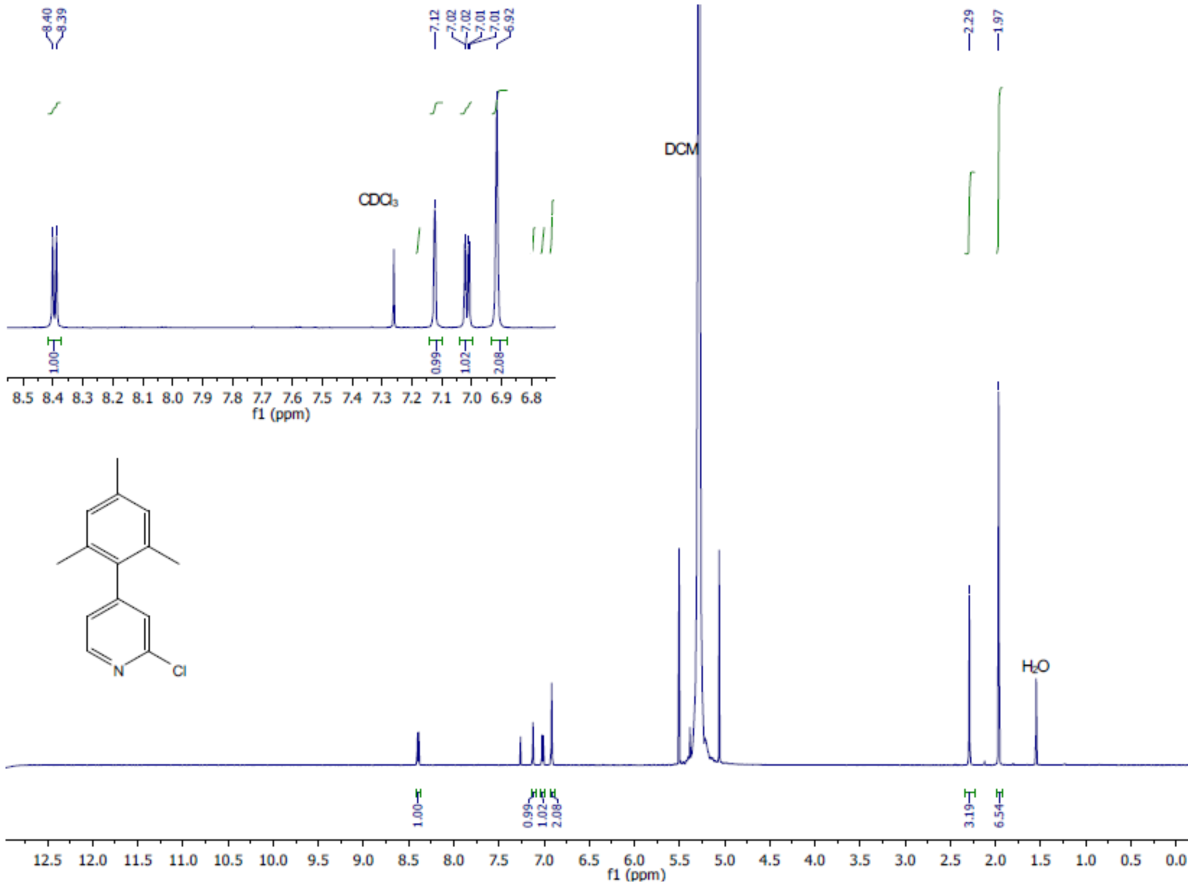

Figure **S4**. ^{1}H NMR spectrum of **2-Chloro-4-(2,4,6-trimethylphenyl)pyridine** in $CDCl_3$.

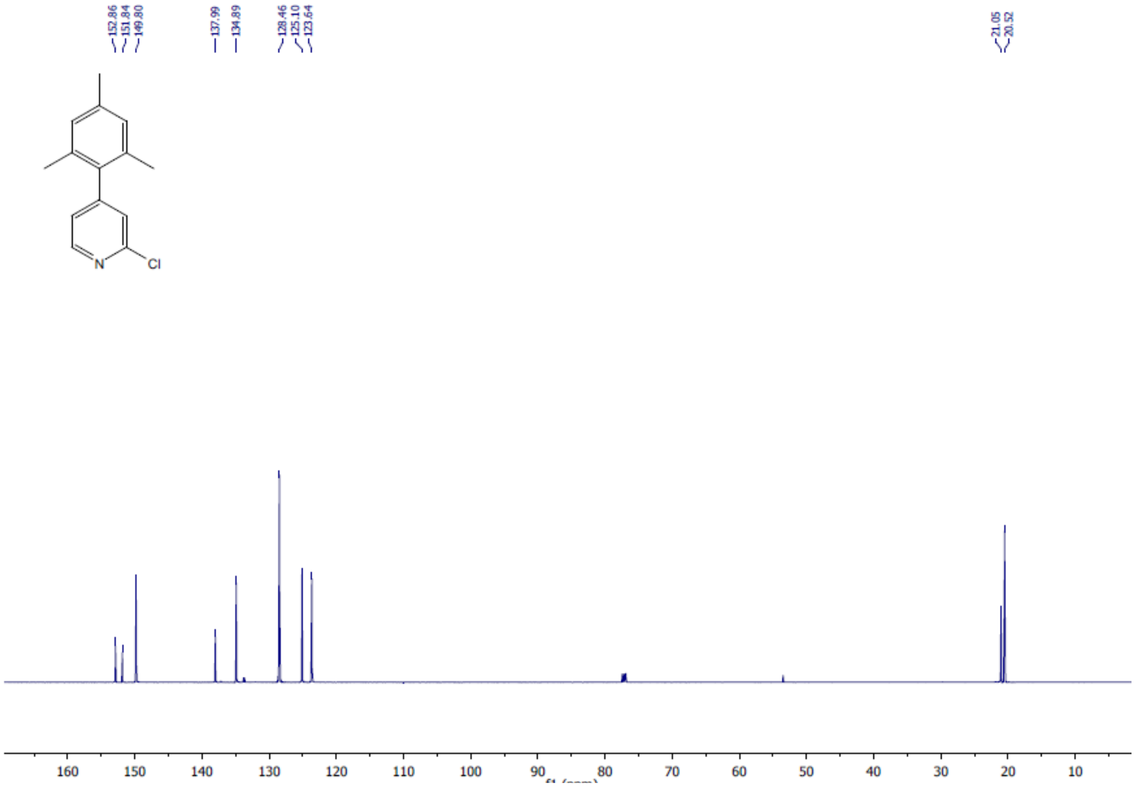

Figure **S5**. ^{13}C NMR spectrum of **2-Chloro-4-(2,4,6-trimethylphenyl)pyridine** in $CDCl_3$.

2-(2,4-Difluorophenyl)-4-(2,4,6-trimethylphenyl)pyridine (MesdFppy).

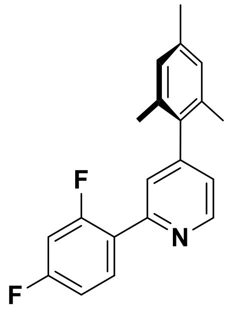

Synthesis of this compound was by a modified procedure to that reported previously.[2] To a round bottom flask was added 2-chloro-4-(2,4,6-trimethylphenyl)pyridine (1.0 equiv.), 2-4-difluorophenyl boronic acid (1.4 equiv.), potassium carbonate (2.5 equiv.), 1,4-dioxane and water (3:1 v/v) to give a solution of ca. 0.75 M. The reaction mixture was degassed by vigorously bubbling N_2 for 20 min. $Pd(PPh_3)_4$ (5 mol%) was added and the reaction mixture sealed. The vessel was then subjected to three cycles of evacuating under vacuum and backfilling with N_2, before heating to reflux 19 h. Upon cooling to room temperature, DCM was added and the layers separated. The organic layer was washed with water, saturated aqueous $NaHCO_3$ and then brine, followed by removal of the solvent *in vacuo* to give the crude material as an oil. Purification by flash column chromatography (silica, hexane/ethyl acetate 9:1) gave the pure compound as a colourless oil (2.88 g). **Yield**: 94%. **R_f:** 0.43 (hexane, 5% ethyl acetate). **^{1}H NMR (500 MHz, $CDCl_3$) δ (ppm):** 8.75 (dd, *J* = 7.5, 1.5 Hz, 1H), 8.08 (dt, *J* = 8.5, 10 Hz, 1H), 7.59 (m, 1H), 7.09 (dd, *J* = 5.0, 1.5 Hz, 1H), 7.02 (tdd, *J* = 10.,0, 3.3, 1.0 Hz, 1H) 6.97 (d, *J* = 1.0 Hz, 2H), 6.90 (dt, *J* = 3.0, 12.5 Hz, 1H), 2.34 (s, 3H), 2.05 (s, 6H). **^{13}C NMR (126 MHz, $CDCl_3$) δ (ppm):** 164.7, 162.0, 159.5, 152.9, 122.8, 150.2, 150.1, 137.8, 136.4, 135.4, 132.4, 132.4, 132.3, 132.3, 128.5, 125.5, 125.4, 123.7, 112.2, 112.1, 111.9, 111.9, 104.8, 104.5, 104.3, 21.2, 20.8. **^{19}F {^{1}H} NMR (471 MHz, $CDCl_3$) δ (ppm):** -109.3 (d, *J* = 9.4 Hz, 1F), -112.7 (d, *J* = 9.4 Hz, 1F). **HR-MS** (APCI$^+$)**: [M+H]$^+$ Calculated:** ($C_{20}H_{17}F_2NH$) 310.1402; **Found:** 310.1402. The characterisation matches that previously reported.[2]

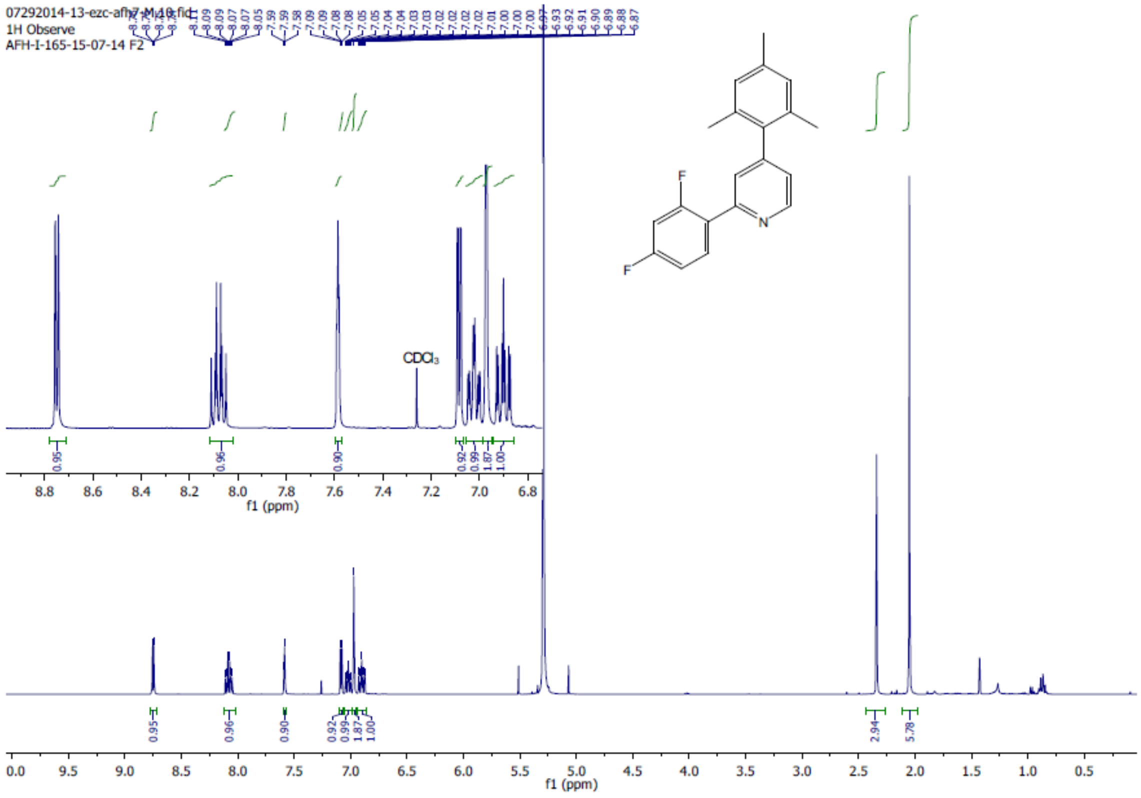

Figure **S6**. $^1H\{^{19}F\}$ NMR spectrum of **2-(2,4-Difluorophenyl)-4-(2,4,6-trimethylphenyl)pyridine** in $CDCl_3$.

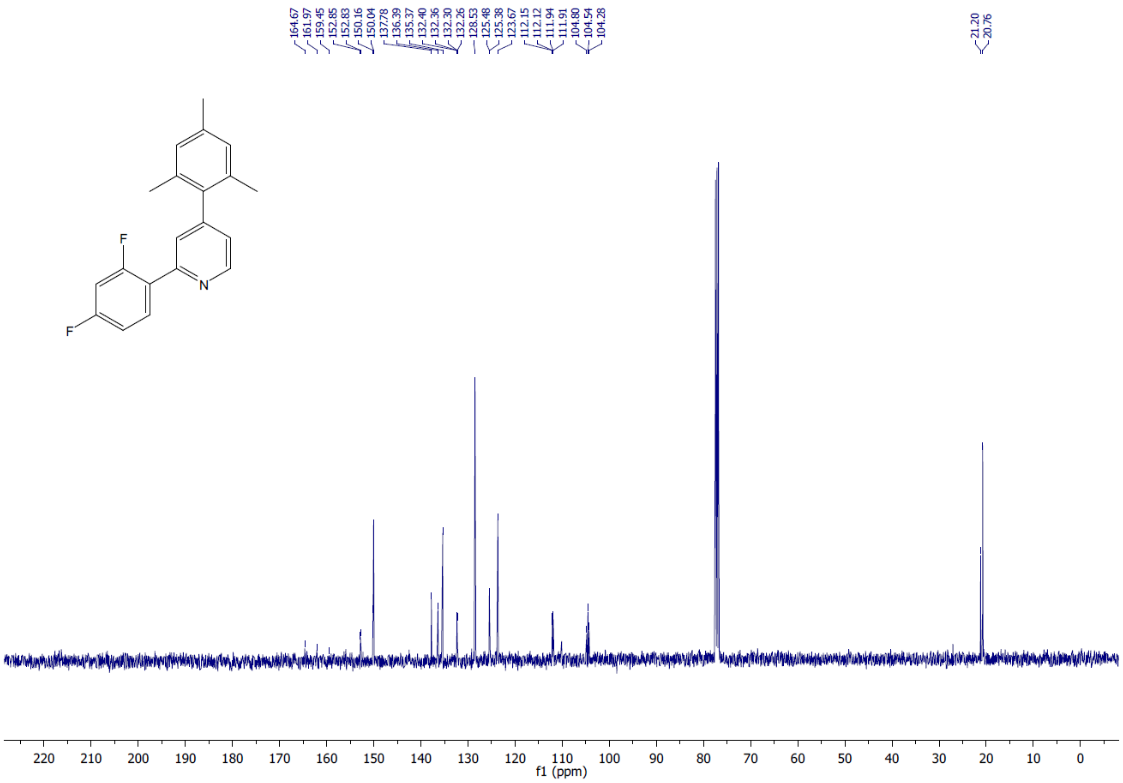

Figure **S7**. ^{13}C NMR spectrum of **2-(2,4-Difluorophenyl)-4-(2,4,6-trimethylphenyl)pyridine** in $CDCl_3$.

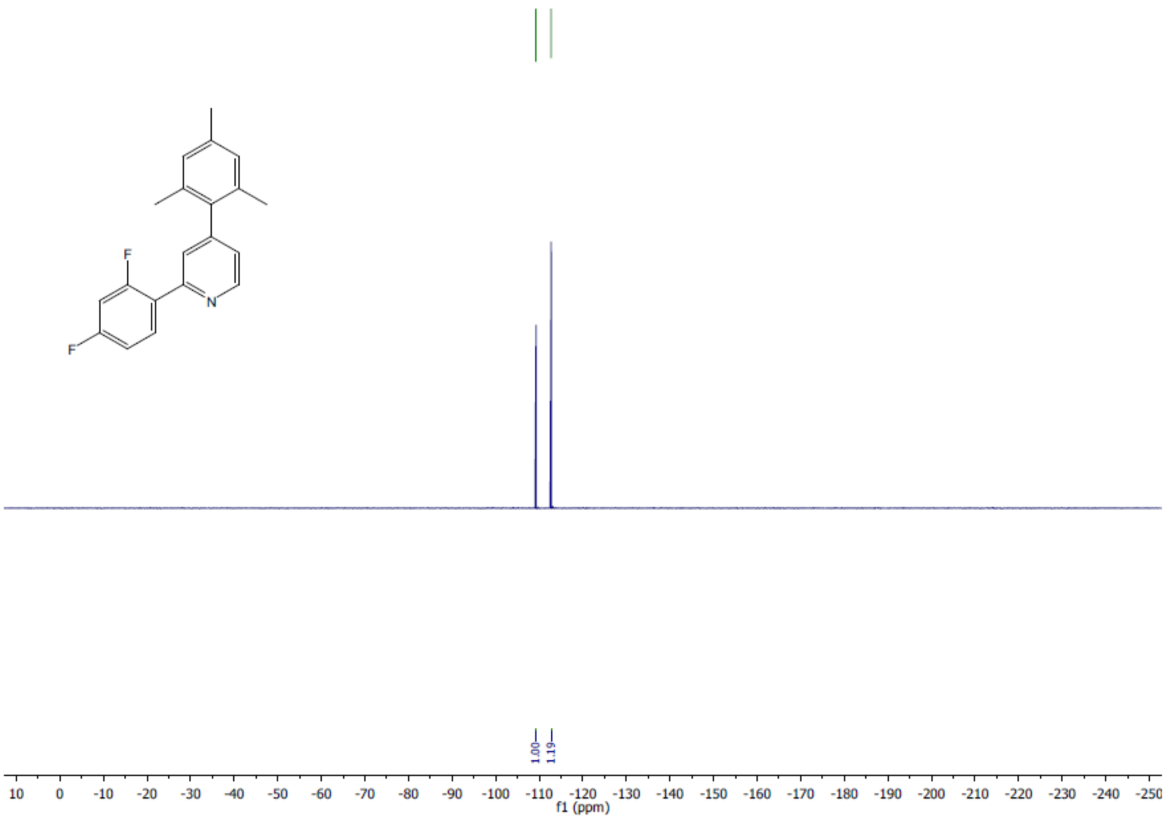

Figure **S7**. $^{19}F\{^{1}H\}$ NMR spectrum of **2-(2,4-Difluorophenyl)-4-(2,4,6-trimethylphenyl)pyridine** in $CDCl_3$.

1*H*,1'*H*-2,2'-biimidazole, (H_2biim).

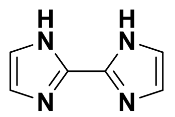

Synthesis of this ligand is as outlined in the literature.[3] To a mixture of ammonium acetate (2.7 equiv.) in distilled water at 40 °C was added dropwise 40% aqueous glyoxal solution (1.0 equiv.) over a period of 3 h to give a concentration of .01 M. The mixture was allowed to stir for a further 5 h at room temperature. The reaction mixture was filtered and washed multiple times with distilled water and acetone to give 8.31 g of a brown crude product. This material was added to ethylene glycol (0.5 M), heated to 150 °C and treated with decolourising carbon. Filtration saw product precipitate immediately, with further washings with distilled water to maximise product precipitation. The product was filtered and dried to give 2.47 g as a cream white powder. **Yield:** 33%. **R_f:** 0.12 (10% MeOH/DCM on silica). **Mp:** 350 - 352 °C. **Litt:** > 300 °C.[7] **^{1}H NMR (500 MHz, DMSO-*d*$_6$) δ (ppm):** 12.67 (s, 2H), 7.14 (s, 2H), 7.00 (s, 2H). **^{13}C NMR (126 MHz, $CDCl_3$) δ (ppm):** 139.8, 128.7, 117.9. The ^{1}H NMR differs from that previously reported but matches that determined by us from a commercial source.[3]

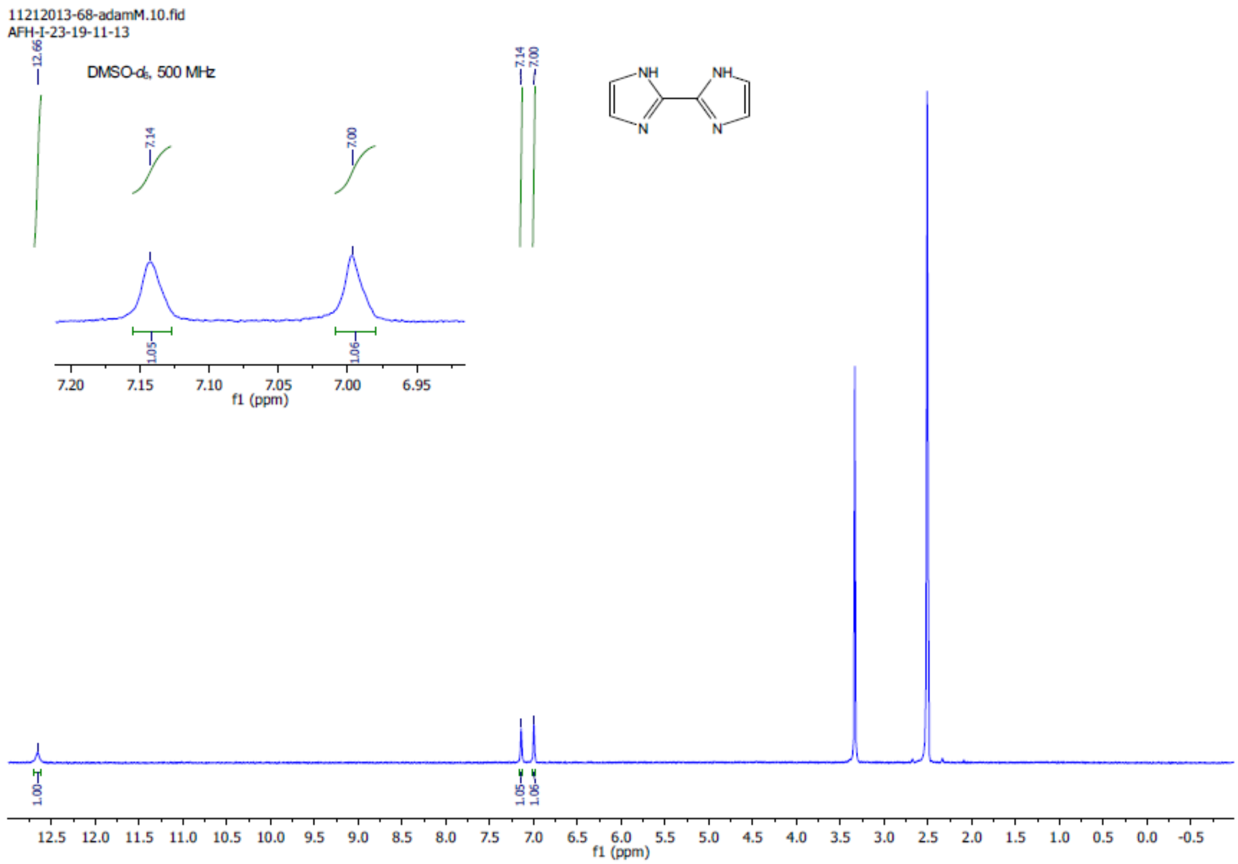

Figure **S8**. ^{1}H NMR spectrum of **1*H*,1'*H*-2,2'-biimidazole (H$_2$biim)** in DMSO-d_6.

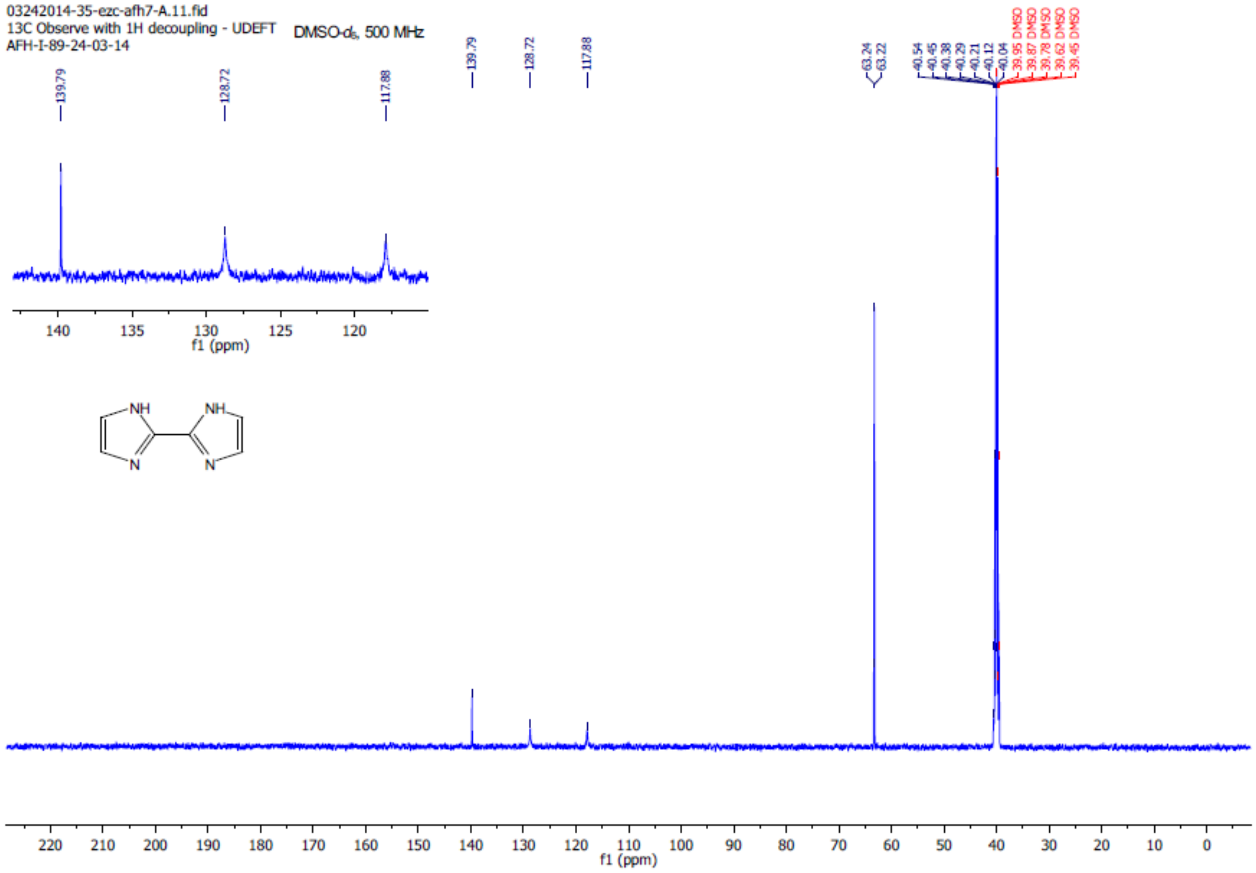

Figure **S9**. ^{13}C NMR spectrum of **1*H*,1'*H*-2,2'-biimidazole (H_2biim)** in DMSO-d_6.

1,1'-(α,α'-*o*-Xylylene)-2,2'-biimidazole (*o*-Xylbiim).

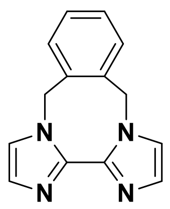

Synthesis of this ligand was as outlined in the literature.[4] To a solution containing α,α'-dibromo-*o*-xylene (1.0 equiv.) in acetonitrile (0.1 M) was added with stirring 1*H*,1'*H*-biimidazole (1.2 equiv.) followed by aqueous sodium hydroxide (5.6 equiv., 35% v/w) solution. The temperature was increased to reflux, where after about 10 min a yellow-brown solution formed. The mixture was maintained at reflux overnight, before being cooled to room temperature. After addition of distilled water the mixture was extracted with multiple times with dichloromethane. The organic fractions were combined, dried over anhydrous magnesium sulfate and then evaporated to dryness under reduced pressure. The crude product was washed with portions of diethyl ether to give an off-white solid. The solid was then redissolved in the minimum of dichloromethane and added to dropwise to stirring diethyl ether, with the formation of a white precipitate, which was collected to afford 0.23 g of the pure compound as a white solid. **Yield:** 35%. **Mp:** 288-291 °C. **Litt:** 284 - 292 °C.[7] **^{1}H NMR (500 MHz, DMSO-*d*$_6$) δ (ppm):** 7.47 (d, *J* = 1.0 Hz, 2H), 7.41 - 7.47 (m, 4H), 7.11 (d, *J* = 0.5 Hz, 2H), 4.97 (s, 4H). **^{13}C NMR (126 MHz, CDCl$_3$) δ (ppm):** 139.4, 133.9, 130.1, 128.9, 128.7, 122.1, 49.0. Characterisation matches that previously reported.[4]

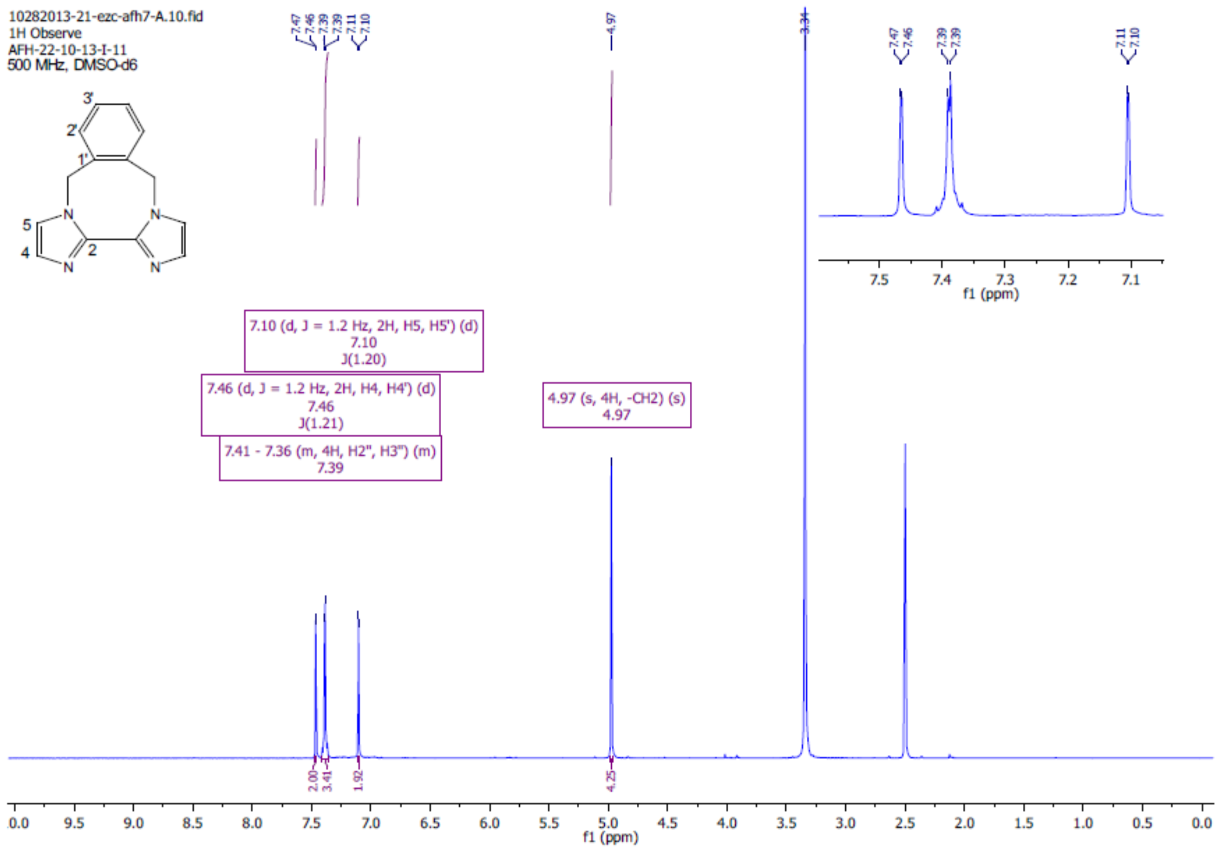

Figure **S10**. ^{1}H NMR spectrum of **1,1'-(α,α'-*o*-Xylylene)-2,2'-biimidazole (*o*Xylbiim)** in DMSO-d_6.

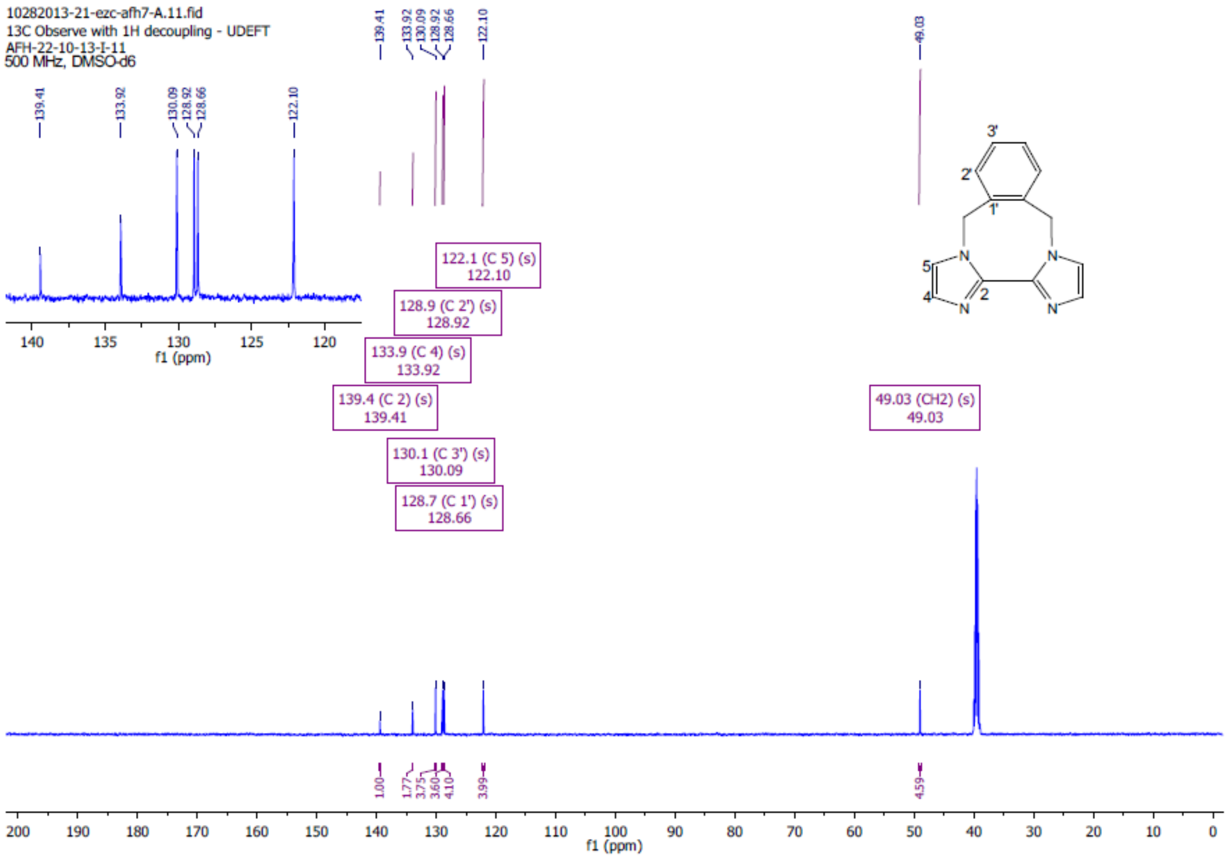

Figure **S11**. ^{13}C NMR spectrum of **1,1'-(α,α'-*o*-Xylylene)-2,2'-biimidazole (*o*Xylbiim)** in DMSO-d_6.

Tetrakis[2-(4',6'-difluorophenyl)-pyridinato-*N*,$C^{2'}$]-bis(*μ*-chloro)diiridium(III), Ir(dFppy)$_2$(*μ*-Cl]$_2$.

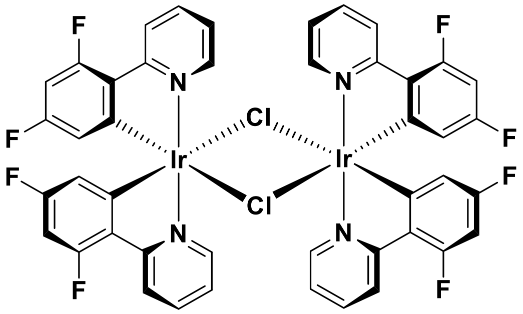

The synthesis of this dimer was a modification of that reported in the literature.[5] A suspension of bis(1,5-cyclooctadiene)diiridium(I) dichloride (1.0 equiv.) in 2-ethoxyethanol was degassed via vigorous nitrogen bubbling. A solution of 2,4-difluorophenylpyridine (4.0 equiv.) in 2-ethoxyethanol (ca. 1.8 M) was added

to the reaction mixture to give a concentration of ca. 0.5 M and the mixture was degassed via nitrogen bubbling again. The reaction mixture was heated to reflux. After 30 min, the reaction mixture turned dark red/black. At 1 h, a yellow precipitate had formed. After 3 h, the reaction mixture was cooled and MeOH was added. The precipitate was filtered, washed with MeOH and acetone, and dried to give the compound as a yellow powder. **Yield:** 46%. **^{1}H NMR (500 MHz, CD_2Cl_2) δ (ppm):** 9.12 (d, *J* = 5.0 Hz, 4H), 8.33 (d, *J* = 8.5 Hz, 4H), 7.88 (t, *J* = 7.0 Hz, 4H), 6.87 (td, *J* = 5.8, 1.0 Hz, 4H), 6.38 (td, *J* = 11.0, 2.0 Hz, 4H) 5.31 (dd, *J* = 9.3, 2.0 Hz, 4H). **^{19}F {^{1}H} NMR (471 MHz, CD_2Cl_2) δ (ppm):** -108.4 (d, *J* = 9.4 Hz, 4F), -110.6 (d, *J* = 9.4 Hz, 4F).

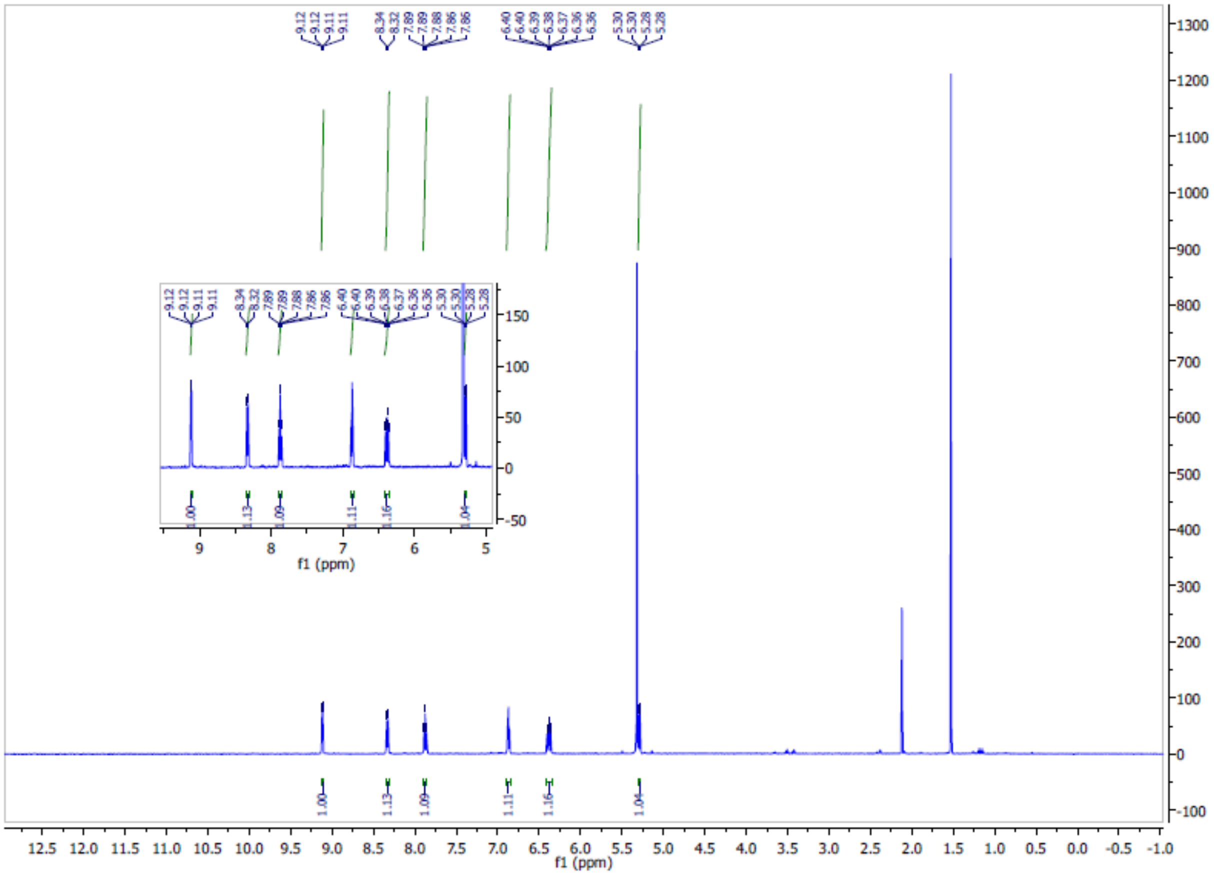

Figure **S12**. ^{1}H NMR spectrum of **Tetrakis[2-(4',6'-difluorophenyl)-pyridinato-*N*,*C*$^{2'}$]-bis(*μ*-chloro)diiridium(III), Ir(dFppy)$_2$(*μ*-Cl]$_2$** in CD_2Cl_2.

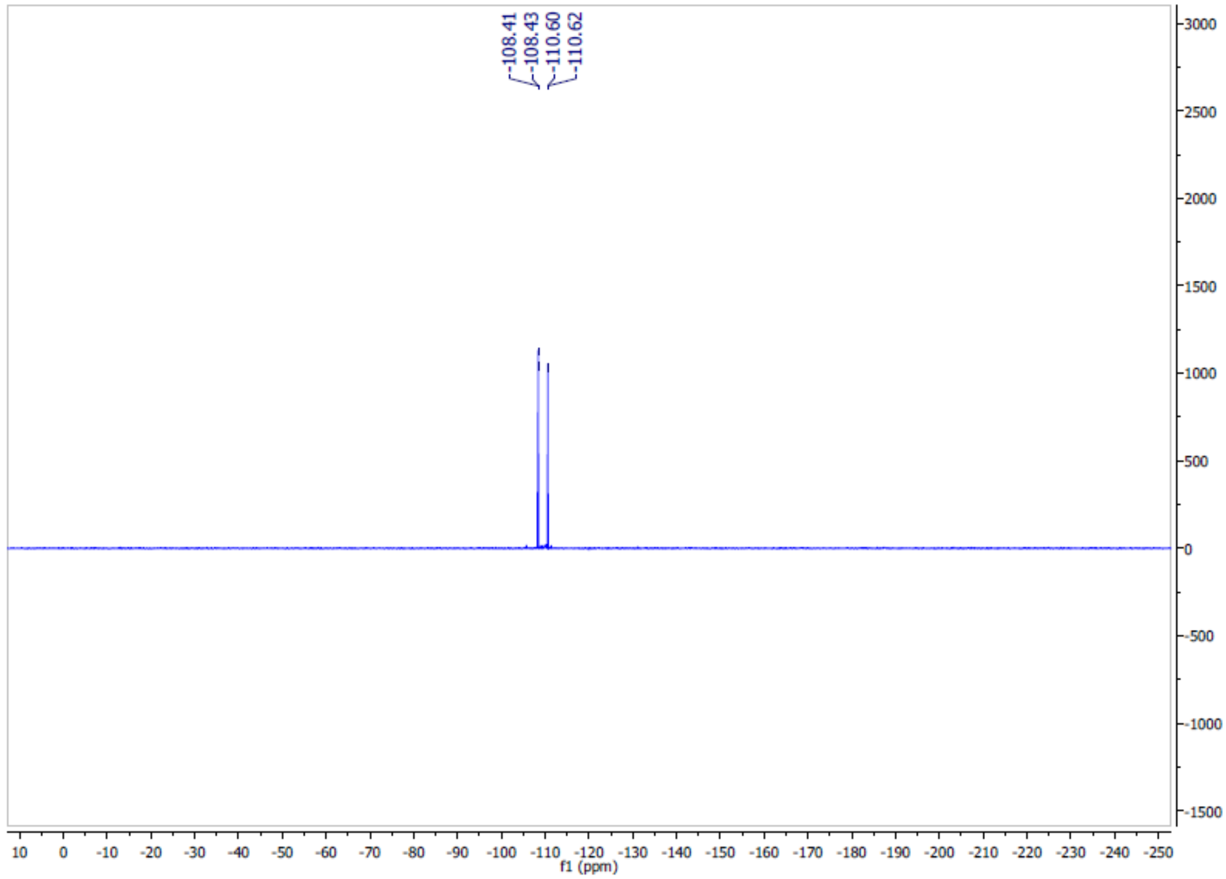

Figure **S13**. ^{19}F NMR spectrum of **Tetrakis[2-(4',6'-difluorophenyl)-pyridinato-*N*,*C*$^{2'}$]-bis(*μ*-chloro)diiridium(III), Ir(dFppy)$_2$(*μ*-Cl]$_2$** in CD_2Cl_2.

Tetrakis[2-(4',6'-difluorophenyl)-4-(2,4,6-trimethylphenyl)pyridinato-*N*,$C^{2'}$]-bis(*μ*-chloro)diiridium(III), Ir(MesdFppy)$_2$(*μ*-Cl]$_2$

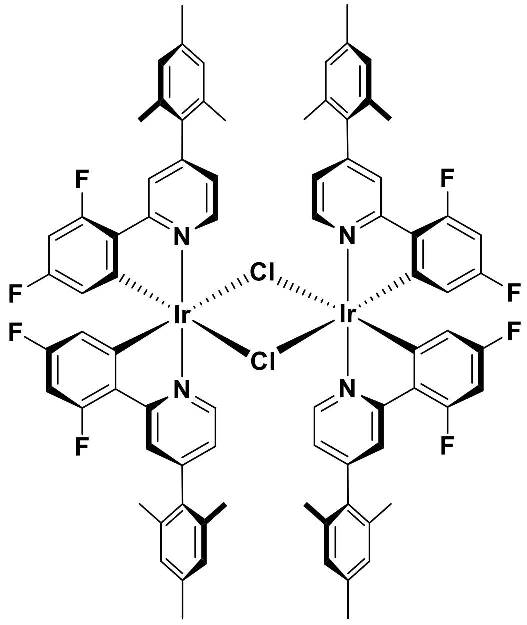

This dimer was prepared analogously to a previous report employing the [Ir(COD)(μ-Cl)]$_2$ dimer as the iridium source.[5] [Ir(COD)(μ-Cl)]$_2$ (1.0 equiv.) and 2-ethoxyethanol were added to a round bottom flask to give a suspension with a concentration of 0.14 M. This mixture was degassed via vigorously bubbling with N_2. In a separate flask, a 2.4 M solution of dFMesppy (4.0 equiv.) in 2-ethoxyethanol was also bubbled vigorously with N_2, before adding to the suspension of [Ir(COD)(μ-Cl)]$_2$ to give an overall concentration of 0.59 M. The mixture was further degassed by three cycles of evacuating under vacuum and backfilling with N_2. The reaction mixture was heated to reflux whereupon it turned a dark red/black. After 1.5 h, a yellow precipitate had formed. After 3 h, the reaction mixture was cooled to room temperature, and concentrated under reduced pressure. Water was added, and the solid was filtered. The solid was washed further with water and then acetone, to give the compound as a yellow powder (0.807 g). **Yield**: 81%. **^{1}H {^{19}F} NMR (400 MHz, CD_2Cl_2) δ (ppm):** 9.57 (d, *J* = 5.6 Hz, 4H), 8.13 (d *J* = 1.2 Hz, 4H), 7.01

(d, J = 10.0 Hz, 8H), 6.89 (dd, J = 6.0, 2.0 Hz, 4H), 6.38 (d, J = 2.4 Hz, 4H), 5.28 (d, J = 2.4 Hz, 4H), 2.38 (s, 12H), 2.12 (s, 12H), 2.10 (s, 12H). **^{19}F {^{1}H} NMR (371 MHz, DMSO-d_6) δ (ppm):** -108.07 (d, J = 11.13 Hz, 4F), -110.22 (d, J = 7.42 Hz, 4F)**.**

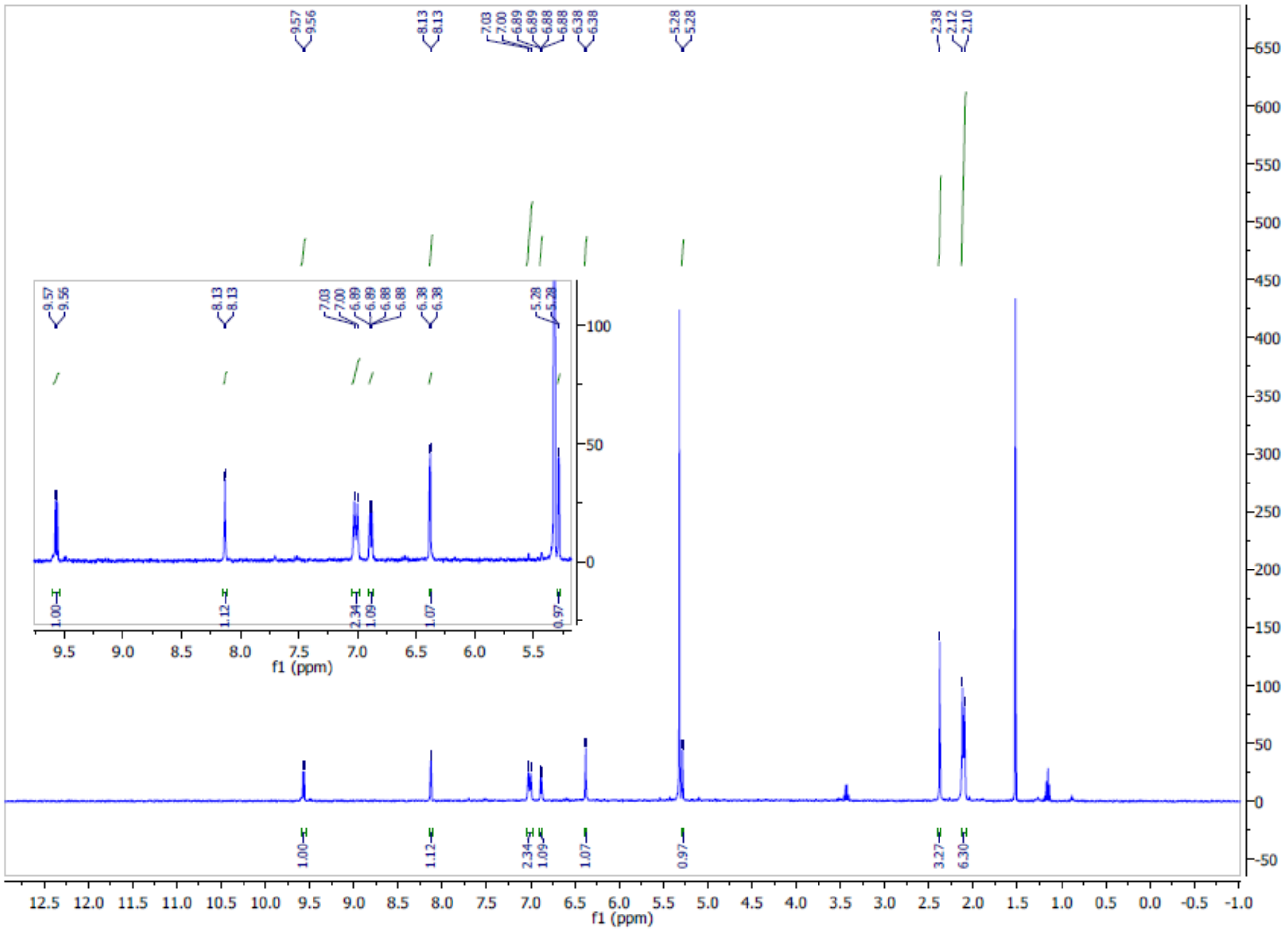

Figure **S14**. ^{1}H NMR spectrum of **Tetrakis[2-(4',6'-difluorophenyl)-4-(2,4,6-trimethylphenyl)pyridinato-*N*,*C*$^{2'}$]-bis(*μ*-chloro)diiridium(III), Ir(MesdFppy)$_2$(*μ*-Cl]$_2$** in CD_2Cl_2.

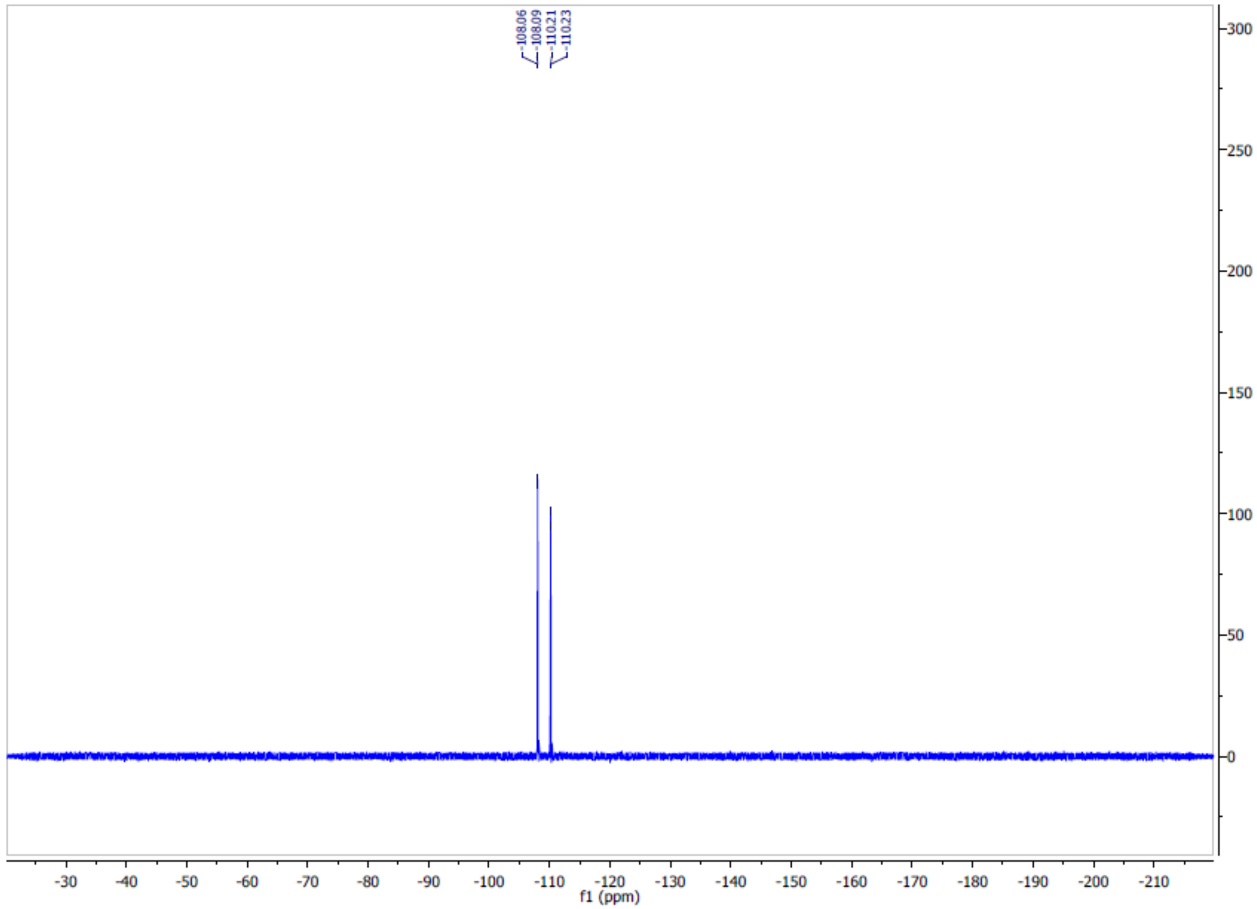

Figure **S15**. ^{19}F NMR spectrum of **Tetrakis[2-(4',6'-difluorophenyl)-4-(2,4,6-trimethylphenyl)pyridinato-*N*,*C*$^{2'}$]-bis(*μ*-chloro)diiridium(III), Ir(MesdFppy)$_2$(*μ*-Cl]$_2$** in CD_2Cl_2.

General procedure for the synthesis of $[(C^\wedge N)_2Ir(N^\wedge N)]PF_6$ complexes. To a round bottom flask containing the appropriate dichloro-bridged iridium dimer (1.0 equiv.) and N^N ligand (2.2 equiv.) were added DCM and MeOH (1:1 v/v) to give a suspension with a concentration of ca. 0.02 M. The mixture was degassed via bubbling with N_2 for 20 minutes, before the reaction vessel was sealed. The reaction mixture was heated to 50 °C for 19 h. Over the course of the reaction the mixture everything dissolved. The solution was cooled to room temperature, and the solvent evaporated. The crude product was purified by flash column chromatography (silica, DCM/MeOH gradient 100:0 to 90:10), and the appropriate fractions were collected and the solvent evaporated. This material was dissolved in the minimum volume of MeOH, and added dropwise to vigorously stirring aqueous NH_4PF_6 (1 g / 10 mL). The fine precipitate that formed was left under continuous stirring for 3 h, until the precipitate had sufficiently aggregated. The material was filtered, washed with water and ether/hexane (1:1 v/v) solution and then dried to afford the final product.

Iridium (III) bis[2-(4',6'-difluorophenyl)-pyridinato-N,C$^{2'}$]-*N,N'*-(4,4'-di-*tert*-butyl-2,2'-bipyridine)hexafluorophosphate: [Ir(dFppy)$_2$(dtbubpy)](PF$_6$).

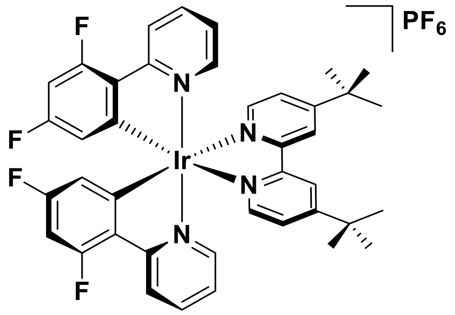

Yellow powder (0.116 g). **Yield**: 72%. **^{1}H {^{19}F} NMR (400 MHz, CD$_2$Cl$_2$) δ (ppm):** 8.33 (d, *J* = 8.0, Hz, 2H), 8.31 (d, *J* = 1.6 Hz, 2H), 7.90 (d, *J* = 5.6 Hz, 2H), 7.85 (td, *J* = 8.0, 1.6 Hz, 2H), 7.50 (d, *J* = 7.5 Hz, 4H), 7.07 (td, *J* = 5.6, 1.2 Hz, 2H), 6.61 (d, *J* = 2.0 Hz, 2H), 5.73 (d, *J* = 2.4 Hz, 2H), 1.44 (s, 18H). **^{13}C {^{1}H} NMR (126 MHz, CD$_2$Cl$_2$) δ (ppm):** 165.16, 165.08, 164.65, 164.59, 163.13, 163.04, 162.96, 162.86, 160.88, 160.78, 156.64, 154.49, 154.44, 150.52, 149.12, 139.52, 128.07, 126.34, 124.25, 124.09, 124.00, 121.79, 114.33, 114.18, 99.54, 99.32, 99.11, 36.10, 30.32. **^{19}F {^{1}H} NMR (371 MHz, CD$_2$Cl$_2$) δ (ppm):** -72.39 (s), -74.27 (s), -106.46 (d, *J* = 11.13 Hz, 2F), -108.98 (d, *J* = 11.13 Hz, 2F). **HR-MS** (FTMS+): **[M-PF$_6$]$^+$ Calculated:** ($C_{40}H_{36}N_4F_4Ir$) 841.2481; **Found:** 841.2502. **Anal.** Calcd for $C_{40}H_{36}N_4F_{10}IrP$ (MW 985.93): C, 48.73; H, 3.68; N, 5.68. Found: C, 48.76; H, 3.79; N, 5.64 (average of two runs).

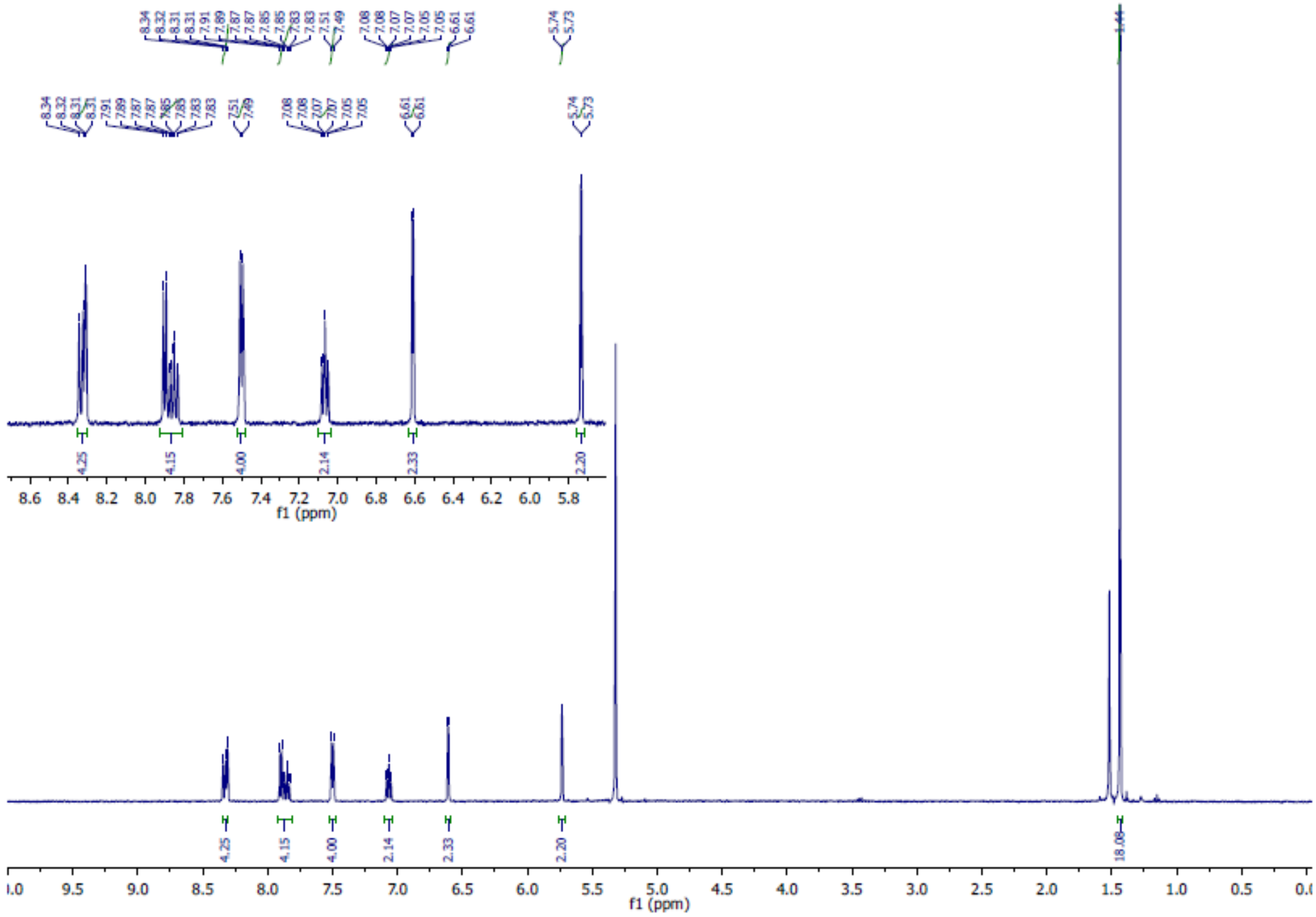

Figure **S16**. ^{1}H NMR spectrum of **Iridium (III) bis[2-(4',6'-difluorophenyl)-pyridinato-N,C$^{2'}$]-*N,N'*-(4,4'-di-*tert*-butyl-2,2'-bipyridine)hexafluorophosphate: [Ir(dFppy)$_2$(dtbubpy)](PF$_6$)** in CD_2Cl_2.

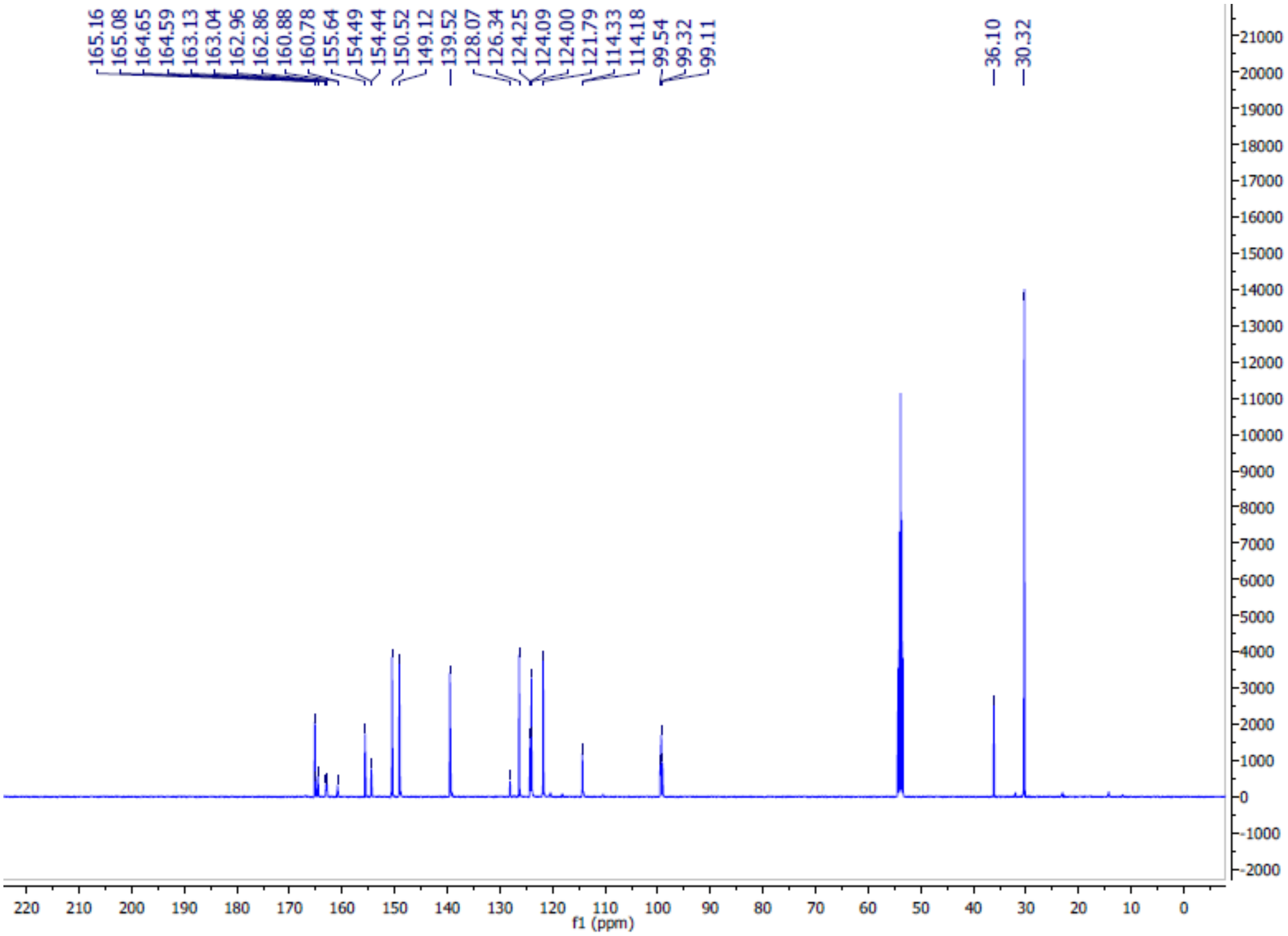

Figure **S17**. ^{13}C NMR spectrum of **Iridium (III) bis[2-(4',6'-difluorophenyl)-pyridinato-N,C$^{2'}$]-*N,N'*-(4,4'-di-*tert*-butyl-2,2'-bipyridine)hexafluorophosphate: [Ir(dFppy)$_2$(dtbubpy)](PF$_6$)** in CD_2Cl_2.

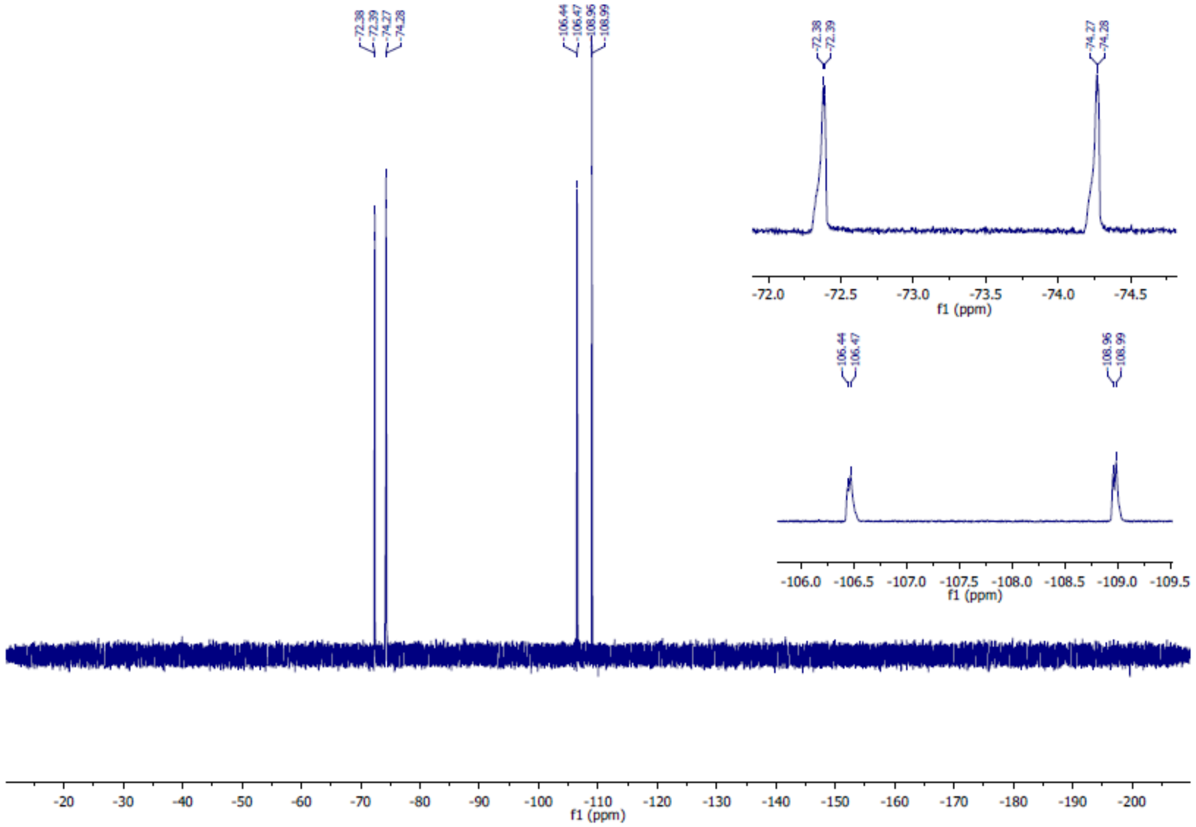

Figure **S18**. ^{19}F NMR spectrum of **Iridium (III) bis[2-(4',6'-difluorophenyl)-pyridinato-N,C$^{2'}$]-*N,N'*-(4,4'-di-*tert*-butyl-2,2'-bipyridine)hexafluorophosphate: [Ir(dFppy)$_2$(dtbubpy)](PF$_6$)** in CD_2Cl_2.

Iridium (III) bis[2-(4',6'-difluorophenyl)-4-(2,4,6-trimethylphenyl)pyridinato-N,C$^{2'}$]-*N,N'*-(4,4'-di-*tert*-butyl-2,2'-bipyridine)hexafluorophosphate: [Ir(MesdFppy)$_2$(dtbubpy)](PF$_6$).

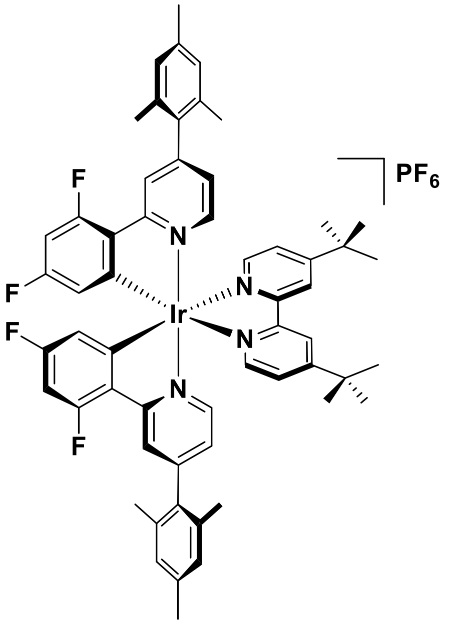

Yellow flakes (0.111 g). **Yield**: 77%. **^{1}H {^{19}F} NMR (400 MHz, CD$_2$Cl$_2$) δ (ppm):** 8.37 (d, *J* = 1.6, Hz, 2H), 8.14 (d, *J* = 1.6 Hz, 2H), 8.02 (d, *J* = 6.0 Hz, 2H), 7.55 (dd, *J* = 5.8, 2.4 Hz, 4H), 6.98 (d, *J* = 16.0 Hz, 4H), 6.91 (dd, *J* = 4.4, 2.0 Hz, 2H), 6.61 (d, *J* = 2.4 Hz, 2H), 5.77 (d, *J* = 2.0 Hz, 2H), 2.32 (s, 6H), 2.11 (s, 6H), 1.94 (s, 6H), 1.46 (s, 18H). **^{13}C {^{1}H} NMR (126 MHz, CD$_2$Cl$_2$) δ (ppm):** 165.25, 165.13, 165.07, 164.72, 164.66, 163.18, 163.08, 162.88, 162.78, 160.80, 160.70,155.86, 154.57, 154.51, 153.56, 150.71, 149.00, 138.82, 135.29, 135.12, 135.09, 128.97, 128.87, 128.23, 126.28, 125.57, 125.46, 125.42, 121.92, 114.25, 114.11, 99.53, 99.32, 99.10, 36.14, 30.35, 21.16, 20.66, 20.53. **^{19}F {^{1}H} NMR (371 MHz, CD$_2$Cl$_2$) δ (ppm):** -72.37 (s), -74.31 (s), -106.38 (d, *J* = 11.13 Hz, 2F), -108.65 (d, *J* = 11.13 Hz, 2F). **HR-MS** (FTMS+): **[M-PF$_6$]$^+$ Calculated:** ($C_{58}H_{56}N_4F_4Ir$) 1077.4069; **Found:** 1077.4041. **Anal.** Calcd for $C_{58}H_{56}N_4F_{10}IrP$ (MW 1222.29): C, 56.99; H, 4.62; N, 4.58. Found: C, 56.85; H, 4.51; N, 4.64 (average of two runs).

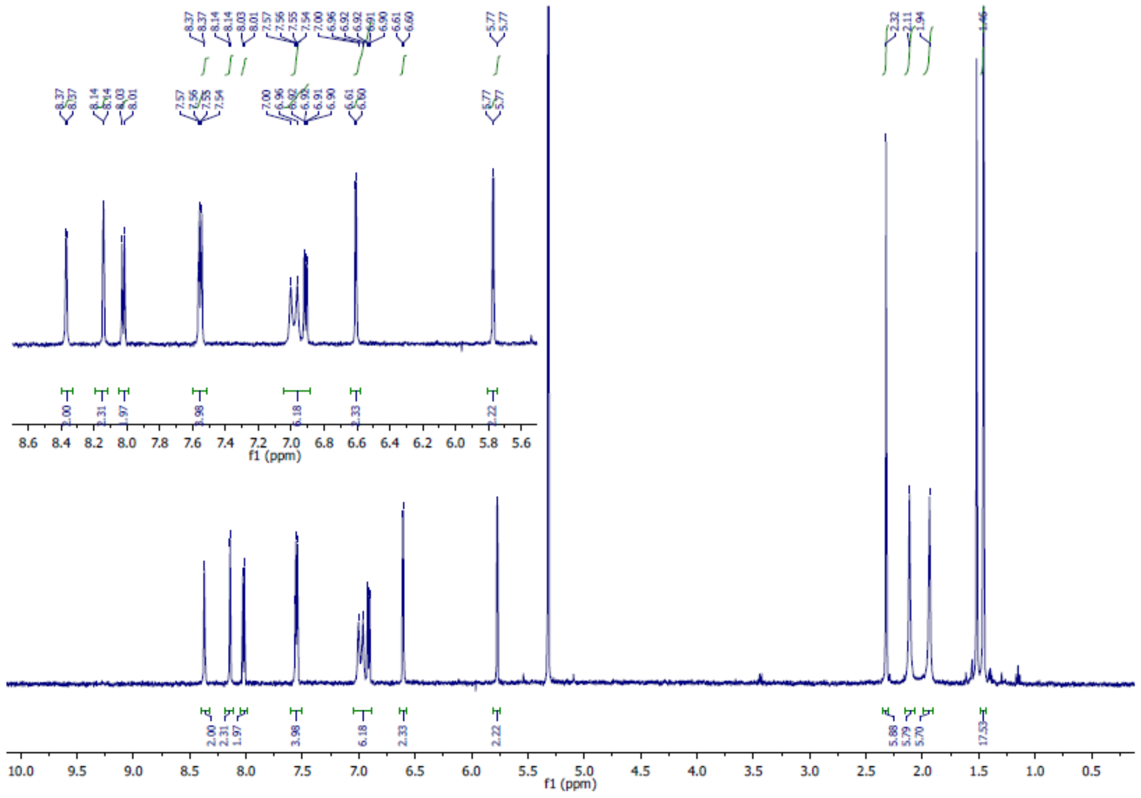

Figure **S19**. ^{1}H NMR spectrum of **Iridium (III) bis[2-(4',6'-difluorophenyl)-4-(2,4,6-trimethylphenyl)pyridinato-N,C$^{2'}$]-*N,N'*-(4,4'-di-*tert*-butyl-2,2'-bipyridine)hexafluorophosphate: [Ir(MesdFppy)$_2$(dtbubpy)](PF$_6$)** in CD_2Cl_2.

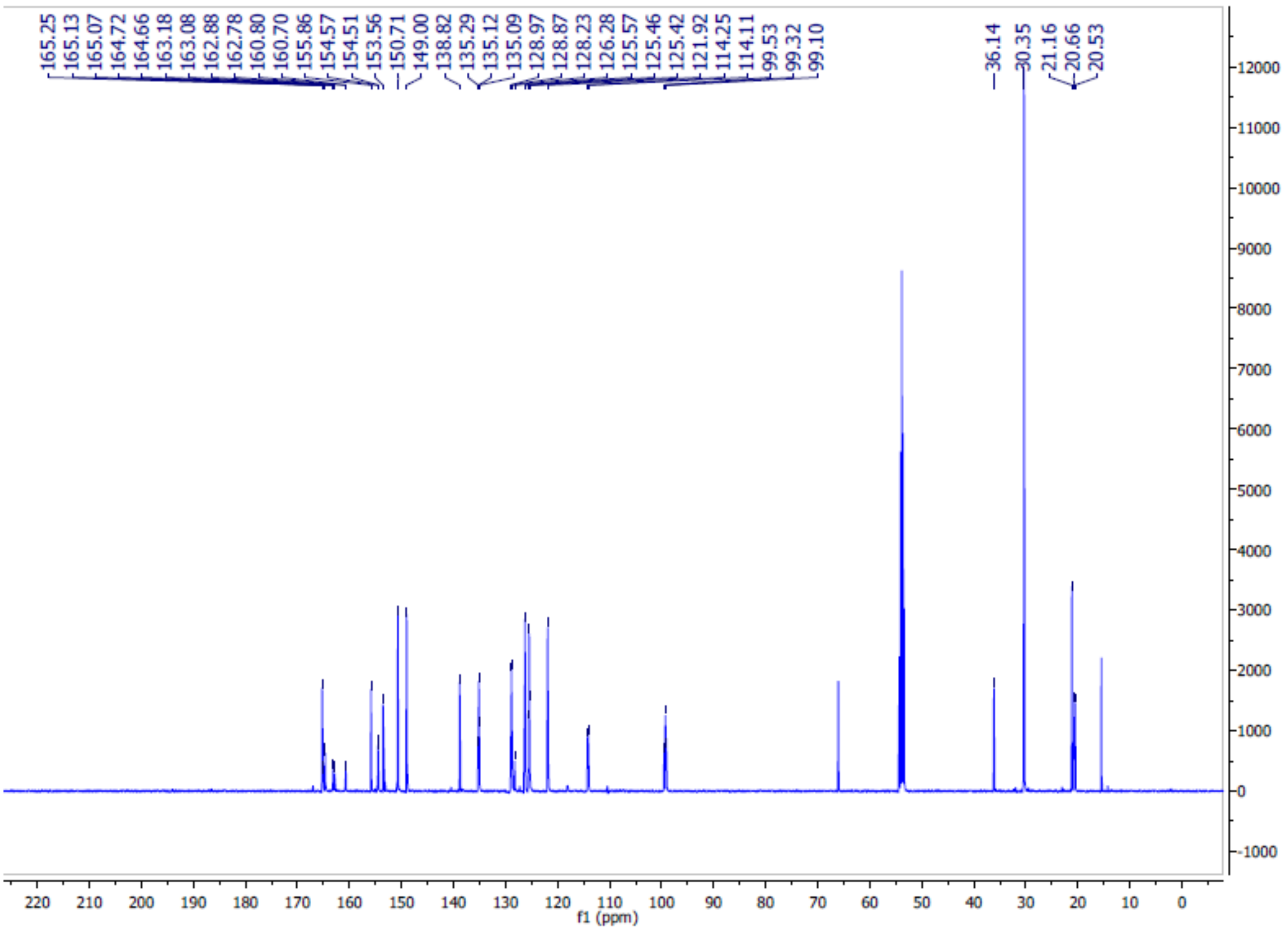

Figure **S20**. ^{13}C NMR spectrum of **Iridium (III) bis[2-(4',6'-difluorophenyl)-4-(2,4,6-trimethylphenyl)pyridinato-N,C$^{2'}$]-*N,N'*-(4,4'-di-*tert*-butyl-2,2'-bipyridine)hexafluorophosphate: [Ir(MesdFppy)$_2$(dtbubpy)](PF$_6$)** in CD_2Cl_2.

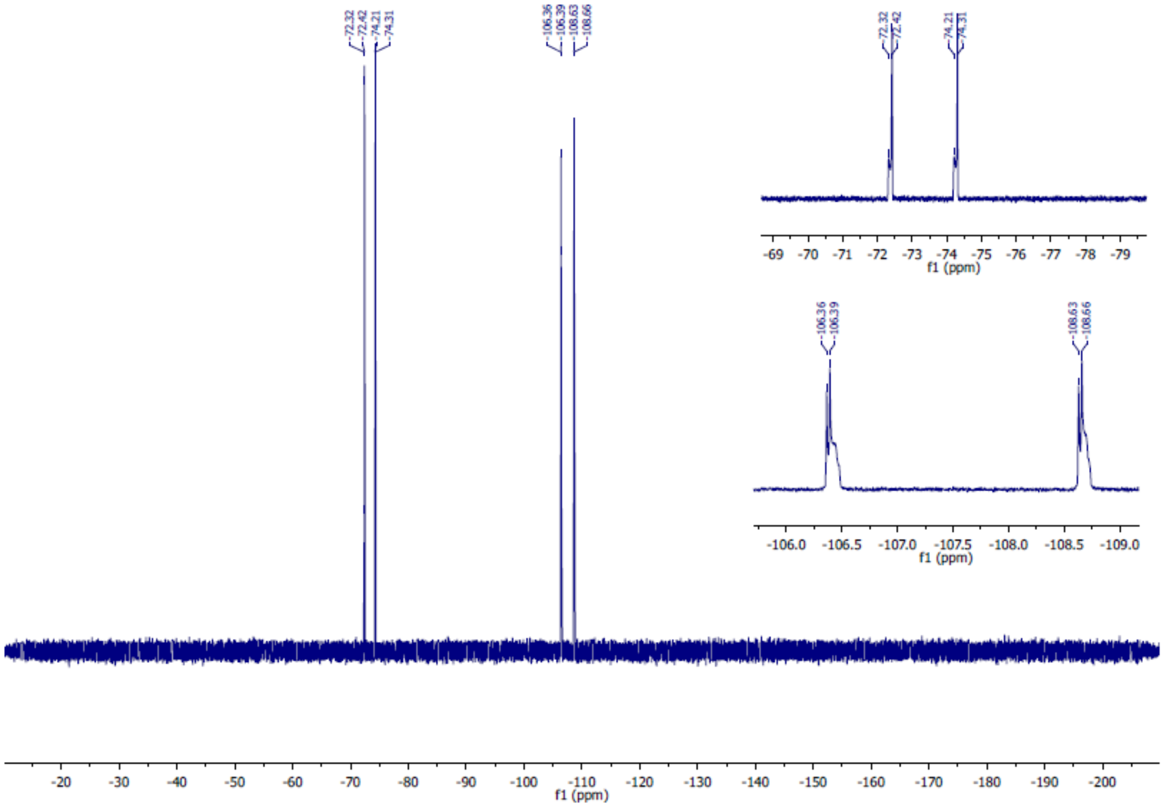

Figure **S21**. ^{19}F NMR spectrum of **Iridium (III) bis[2-(4',6'-difluorophenyl)-4-(2,4,6-trimethylphenyl)pyridinato-N,C$^{2'}$]-*N,N'*-(4,4'-di-*tert*-butyl-2,2'-bipyridine)hexafluorophosphate: [Ir(MesdFppy)$_2$(dtbubpy)](PF$_6$)** in CD_2Cl_2.

Iridium (III) bis[2-(4',6'-difluorophenyl)-4-(2,4,6-trimethylphenyl)pyridinato-N,C$^{2'}$]-*N,N'*-(1,1'-(α,α'-*o*-Xylylene)-2,2-biimidazole)hexafluorophosphate: [Ir(MesdFppy)$_2$(*o*-Xylbiim)](PF$_6$).

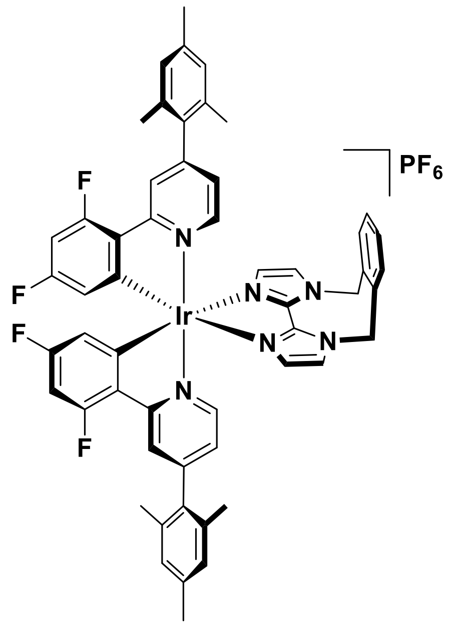

Yellow powder (0.094 g). **Yield**: 66%. **^{1}H {^{19}F} NMR (500 MHz, DMSO-d_6, 372 K) δ (ppm):** 7.91 (s, 2H), 7.80 (s, 2H), 7.62 (t, J = 6.0 Hz, 2H), 7.46 (dd, J = 5.5, 3.5 Hz, 2H), 7.05 – 6.94 (m, 8H), 6.75 (td, J = 11.0, 2.0 Hz, 2H), 6.62 (s, 2H), 5.88 (s, 4H), 5.75 (dd, J = 8.8, 1.5 Hz, 2H), 2.31 (s, 6H), 1.99 (s, 12H). **^{13}C {^{1}H} NMR (126 MHz, CD$_2$Cl$_2$, 298 K) δ (ppm):** 164.61, 164.55, 152.84, 152.65, 149.81, 148.74, 138.60, 138.56, 135.57, 135.36, 135.07, 134.09, 133.88, 131.54, 131.36, 128.81, 127.92, 127.78, 126.00, 125.71, 125.50, 124.85, 124.96, 124.62, 114.37, 114.20, 114.05, 98.75, 98.54, 98.33, 51.39, 51.31, 21.16, 20.64 **^{19}F {^{1}H} NMR (371 MHz, CD$_2$Cl$_2$) δ (ppm):** -71.97 (s), -73.86 (s), -107.70 (d, J = 9.65 Hz, 1F), -108.36 (d, J = 9.65 Hz, 1F), -109.82 – -109.98 (m, 2F)**. HR-MS (**FTMS+**): [M-PF$_6$]$^+$ Calculated:** (C$_{54}$H$_{44}$N$_6$F$_4$Ir) 1045.3191; **Found:** 1045.3160. **Anal.** Calcd for C$_{54}$H$_{44}$N$_6$F$_{10}$IrP (MW 1190.16): C, 54.50; H, 3.73; N, 7.06 Found: C, 54.85; H, 4.11; N, 7.46 (average of two runs).

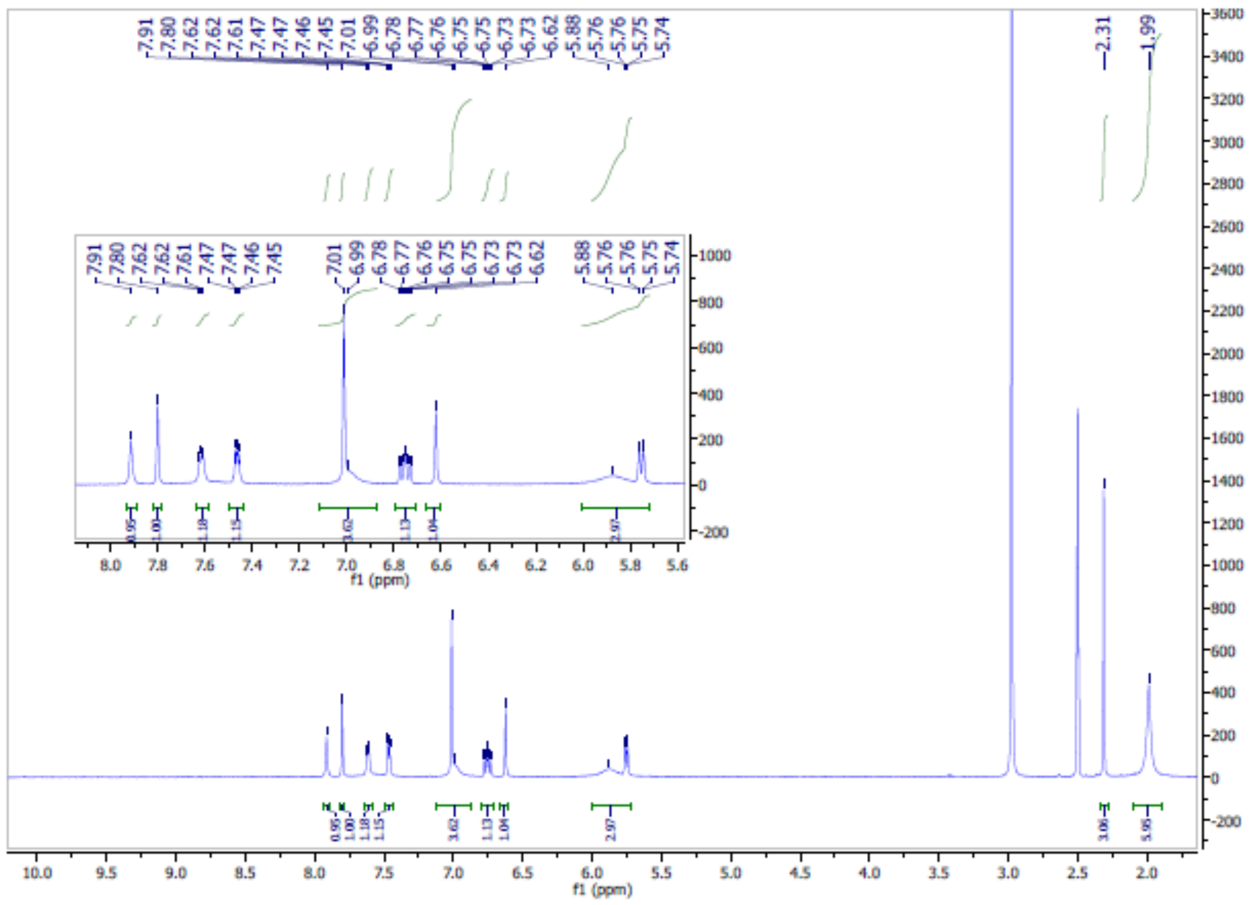

Figure **S22**. ^{1}H NMR spectrum of **Iridium (III) bis[2-(4',6'-difluorophenyl)-4-(2,4,6-trimethylphenyl)pyridinato-N,C$^{2'}$]-*N,N'*-(1,1'-(α,α'-*o*-Xylylene)-2,2-biimidazole)hexafluorophosphate, [Ir(MesdFppy)$_2$(*o*-Xylbiim)](PF$_6$),** in DMSO-d_6 at 378 K.

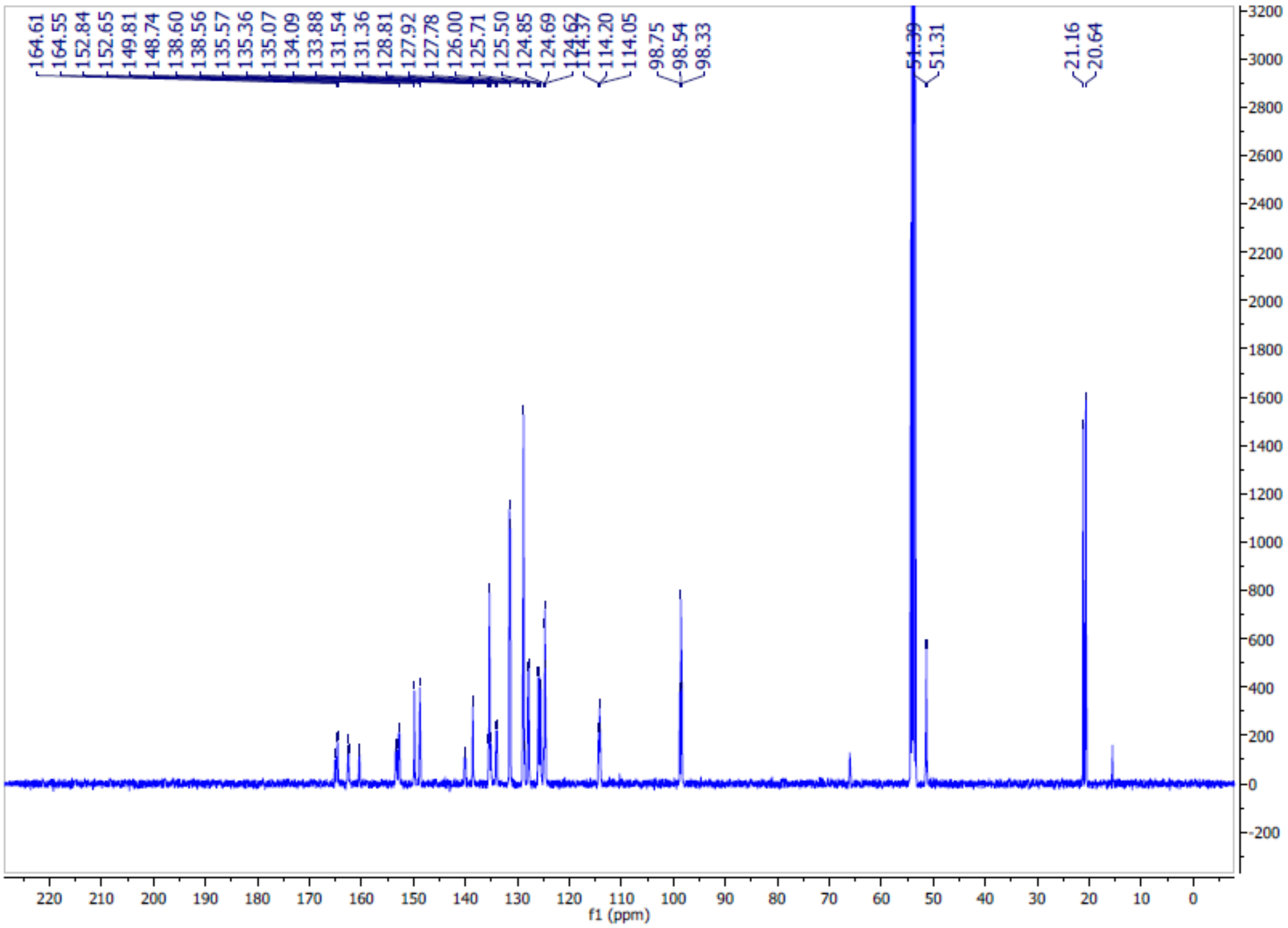

Figure **S23**. ^{13}C NMR spectrum of **Iridium (III) bis[2-(4',6'-difluorophenyl)-4-(2,4,6-trimethylphenyl)pyridinato-N,C$^{2'}$]-*N,N'*-(1,1'-(α,α'-*o*-Xylylene)-2,2-biimidazole)hexafluorophosphate, [Ir(MesdFppy)$_2$(*o*-Xylbiim)](PF$_6$),** in CD_2Cl_2.

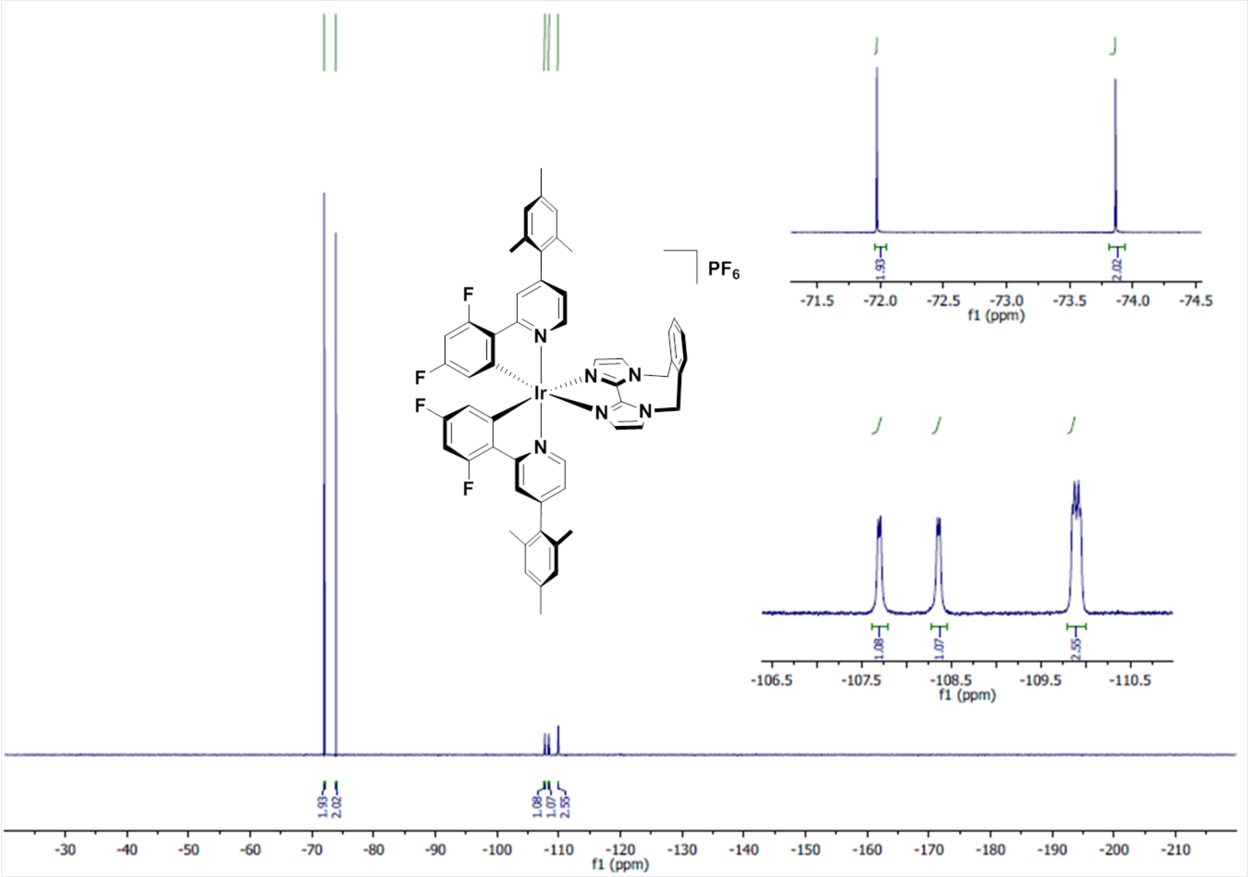

Figure **S24**. ^{19}F NMR spectrum of **Iridium (III) bis[2-(4',6'-difluorophenyl)-4-(2,4,6-trimethylphenyl)pyridinato-N,C$^{2'}$]-*N,N'*-(1,1'-(α,α'-*o*-Xylylene)-2,2-biimidazole)hexafluorophosphate, [Ir(MesdFppy)$_2$(*o*-Xylbiim)](PF$_6$),** in CD_2Cl_2.

Photophysical measurements. All samples were prepared in HPLC grade acetonitrile (MeCN) with varying concentrations on the order of μM. Absorption spectra were recorded at RT using a Shimadzu UV-1800 double beam spectrophotometer. Molar absorptivity determination was verified by linear least-squares fit of values obtained from at least three independent solutions at varying concentrations with absorbance ranging from 1.26×10^{-4} to 3.43×10^{-5} M.

The sample solutions for the emission spectra were prepared in HPLC grade MeOH and degassed via three freeze-pump-thaw cycles. Steady state emission and time-resolved emission spectra were recorded at 298 K using an Edinburgh Instruments F980. All samples for steady state measurements were excited at 360 nm while samples for time-resolved measurements were excited at 378 nm using a PDL 800-D pulsed diode laser. Emission quantum yields were determined using the optically dilute method.[6] A stock solution with absorbance of ca. 0.5 was prepared and then four dilutions were prepared with dilution factors of 5, 6.6, 10 and 20 to obtain solutions with absorbances of ca. 0.1 0.075, 0.05 and 0.025, respectively. The Beer-Lambert law was found to be linear at the concentrations of the solutions. The emission spectra were then measured after the solutions were degassed by nitrogen purging for fifteen minutes per sample prior to spectrum acquisition. For each sample, linearity between absorption and emission intensity was verified through linear regression analysis and additional measurements were acquired until the Pearson regression factor (R^2) for the linear fit of the data set surpassed 0.9. Individual relative quantum yield values were calculated for each solution and the values reported represent the slope value. The equation $\Phi_s = \Phi_r(A_r/A_s)(I_s/I_r)(n_s/n_r)_2$ was used to calculate the relative quantum yield of each of the sample, where Φ_r is the absolute quantum yield of the reference, n is the refractive index of the solvent, A is the absorbance at the excitation

wavelength, and I is the integrated area under the corrected emission curve. The subscripts s and r refer to the sample and reference, respectively. A solution of quinine sulfate in 0.5 M H_2SO_4 (Φr = 54.6%) was used as the external reference.[7]

Samples for solid-state measurements were prepared by dip-coating a quartz substrate in a concentrated solution (5 mg / 1 mL) and drying. Neat films were prepared from MeCN solution and doped films were prepared from DCM solution, with 5 wt% of complex in PMMA. Steady state emission and time-resolved emission spectra were recorded at 298 K using an Edinburgh Instruments F980. All samples for steady state measurements were excited at 360 nm while samples for time-resolved measurements were excited at 378 nm using a PDL 800-D pulsed diode laser, and were recorded under air. Quantum yields were measured using an integrating sphere, under a nitrogen atmosphere.

Electrochemistry measurements. Cyclic voltammetry (CV) measurements were performed on an Electrochemical Analyzer potentiostat model 600D from CH Instruments. Solutions for cyclic voltammetry were prepared in MeCN and degassed with MeCN-saturated nitrogen bubbling for about 10 min prior to scanning. Tetra(*n*-butyl)ammoniumhexafluorophosphate ($TBAPF_6$; ca. 0.1 M in MeCN) was used as the supporting electrolyte. A Pt wire was used as the pseudoreference electrode; a Pt wire coil was used as the counter electrode and a Pt disk electrode was used for the working electrode. The redox potentials are reported relative to a saturated calomel electrode (SCE) electrode with a ferrocenium/ferrocene (Fc^+/Fc) redox couple as an internal reference (0.38 V vs SCE).[8]

X-ray crystallography

Single crystals were grown by vapour diffusion of diethyl ether (**1** and **3**) or di*iso*propyl ether (**2**) into concentrated acetonitrile solutions. Data for **1** and **3** were collected at either 173 K (**1**) or 93 K (**3**) by using a Rigaku FR-X Ultrahigh brilliance Microfocus RA generator/confocal optics and Rigaku XtaLAB P200 system, with Mo Kα radiation (λ = 0.71075 Å). Data for compound **2** was collected at 173 K by using a Rigaku MM-007HF High brilliance RA generator/confocal optics and Rigaku XtaLAB P100 system, with Cu Kα radiation (λ = 1.54187 Å). Intensity data were collected using ω steps (**1** and **3**), or ω and φ steps (**2**) accumulating area detector images spanning at least a hemisphere of reciprocal space. All data were corrected for Lorentz polarization effects. A multiscan absorption correction was applied by using CrystalClear.[9] Structures were solved by Patterson (PATTY)[10] or direct (SIR97)[11] methods and refined by full-matrix least-squares against F[12] (SHELXL-2013).[13] Non-hydrogen atoms were refined anisotropically, and hydrogen atoms were refined using a riding model. All calculations were performed using the CrystalStructure interface.[14]

Table S1. Crystal Data and Structure Refinement.

	**1**	**2**	**3**
empirical formula	$C_{40}H_{36}F_{10}IrN_4P$	$C_{64}H_{70}F_{10}IrN_4OP$	$C_{58}H_{54}F_{10}IrN_6OP$
fw	985.93	1324.46	1264.28
crystal description	green, platelet	yellow, platelet	yellow, prism
crystal size [mm^3]	0.12×0.03×0.01	0.32×0.05×0.01	0.10×0.10×0.02
temp [K]	173	173	93
space group	$P2_1/c$	$P\bar{1}$	$C2/c$
a [Å]	10.4212(16)	11.239(4)	45.7191(10)
b [Å]	15.6794(18)	16.275(5)	9.4584(17)
c [Å]	23.613(3)	20.538(6)	33.211(7)
α [°]		112.85(2)	
β [°]	100.008(4)	94.28(2)	125.688(3)
γ [°]		98.581(18)	
vol [Å]^3	3799.6(9)	3387(2)	11664(3)
Z	4	2	8
ρ (calc) [g/cm^3]	1.723	1.299	1.440
μ [mm^{-1}]	3.651	4.525	2.398
F(000)	1944	1344	5072
reflns collected	44863	34115	44211
independent reflns (R_{int})	6983 (0.1495)	11900 (0.1100)	10577 (0.0697)
data/restraints/params	6983/0/511	11900/39/730	10577/0/703
GOF on F^2	0.998	1.079	1.210
R_1 [$I > 2\sigma(I)$]	0.0959	0.1060	0.0772
wR_2 (all data)	0.1340	0.3111	0.2938
largest diff. peak/hole [e/Å^3]	2.30/–0.95	3.65/–1.53	5.73/–2.43

References

(1). Y. You and S. Y. Park, *J. Am. Chem. Soc.*, 2005, **127**, 12438-12439.

(2). V. N. Kozhevnikov, Y. Zheng, M. Clough, H. A. Al-Attar, G. C. Griffiths, K. Abdullah, S. Raisys, V. Jankus, M. R. Bryce and A. P. Monkman, *Chem. Mater.*, 2013, **25**, 2352-2358.

(3). J.-C. Xiao and J. n. M. Shreeve, *J. Org. Chem.*, 2005, **70**, 3072-3078.

(4). H. V. Phan, P. Chakraborty, M. Chen, Y. M. Calm, K. Kovnir, L. K. Keniley, Jr., J. M. Hoyt, E. S. Knowles, C. Besnard, M. W. Meisel, A. Hauser, C. Achim and M. Shatruk, *Chem. Eur. J.*, 2012, **18**, 15805-15815.

(5). E. Baranoff, B. F. E. Curchod, J. Frey, R. Scopelliti, F. Kessler, I. Tavernelli, U. Rothlisberger, M. Grätzel and M. K. Nazeeruddin, *Inorg. Chem.*, 2011, **51**, 215-224.

(6). (a) G. A. Crosby and J. N. Demas, *J. Phys. Chem.*, 1971, **75**, 991-1024; (b) S. Fery-Forgues and D. Lavabre, *J. Chem. Educ.*, 1999, **76**, 1260.

(7). W. H. Melhuish, *J. Phys. Chem.*, 1961, **65**, 229-235.

(8). V. V. Pavlishchuk and A. W. Addison, *Inorg. Chim. Acta*, 2000, **298**, 97-102.

(9). *CrystalClear-SM Expert v. 2.1;* , Rigaku Americas, Rigaku Americas, The Woodlands, Texas, USA and Rigaku Corporation, Tokyo, Japan, 2010-2014.
(10). P. T. Beurskens, G. Beurskens, R. de Gelder, S. Garcia-Granda, R. O. Gould, R. Israel and J. M. M. Smits, Crystallography Laboratory, University of Nijmegen, The Netherlands, , 1999.
(11). A. Altomare, M. C. Burla, M. Camalli, G. L. Cascarano, C. Giacovazzo, A. Guagliardi, A. G. G. Moliterni, G. Polidori and R. Spagna, *J. Appl. Crystallogr.*, 1999, **32**, 115-119.
(12). M. C. Burla, R. Caliandro, M. Camalli, B. Carrozzini, G. L. Cascarano, L. De Caro, C. Giacovazzo, G. Polidori and R. Spagna, *J. Appl. Crystallogr.*, 2005, **38**, 381-388.
(13). G. Sheldrick, *Acta Crystallogr., Sect. C: Cryst. Struct. Commun.*, 2015, **71**, 3-8.
(14). *CrystalStructure v4.1* Rigaku Americas, Rigaku Americas, The Woodlands, Texas, USA and Rigaku Corporation, Tokyo, Japan, 2013.
